# Predicting climate change effects on wetland ecosystem services using species distribution modeling and plant functional traits

**DOI:** 10.1007/s13280-014-0593-9

**Published:** 2015-01-09

**Authors:** Helen Moor, Kristoffer Hylander, Jon Norberg

**Affiliations:** 1 Stockholm Resilience Centre, Stockholm University, 106 91 Stockholm, Sweden; 2 Department of Ecology, Environment and Plant Sciences, Stockholm University, 106 91 Stockholm, Sweden

**Keywords:** Functional traits, Ecosystem services, Climate change, Species distribution modeling, Wetlands, Sweden

## Abstract

Wetlands provide multiple ecosystem services, the sustainable use of which requires knowledge of the underlying ecological mechanisms. Functional traits, particularly the community-weighted mean trait (CWMT), provide a strong link between species communities and ecosystem functioning. We here combine species distribution modeling and plant functional traits to estimate the *direction* of change of ecosystem processes under climate change. We model changes in CWMT values for traits relevant to three key services, focusing on the regional species pool in the Norrström area (central Sweden) and three main wetland types. Our method predicts proportional shifts toward faster growing, more productive and taller species, which tend to increase CWMT values of specific leaf area and canopy height, whereas changes in root depth vary. The predicted changes in CWMT values suggest a potential increase in flood attenuation services, a potential increase in short (but not long)-term nutrient retention, and ambiguous outcomes for carbon sequestration.

**Electronic supplementary material:**

The online version of this article (doi:10.1007/s13280-014-0593-9) contains supplementary material, which is available to authorized users.

## Introduction

Ecosystem services (ES) lie at the heart of interactions between society and nature. Wetlands are natural assets that can deliver ES from local to regional scales (Barbier 2011). Due to their unique characteristics, they influence global biogeochemical and hydrological cycles in spite of their scattered nature (Mitsch and Gosselink 2007). In Sweden, wetlands are a conservation priority, and in spite of large losses due to drainage and land use change, an estimated 20 % of total land area remains as wetlands (Gunnarsson and Löfroth 2009).

ES are defined as the benefits society obtains, directly or indirectly, from ecosystems (Hooper et al. 2005). ES are thus ecosystem properties or processes that are recognized, utilized, and valued by people. Wetlands are appreciated for fodder production, water flow and quality regulation, climate regulation, and recreation (SEPA 2012). While acknowledging societal dynamics that influence ES demands and usage, adopting a mechanistic, process-based approach to understand factors underlying ES delivery is critical to provide relevant management recommendations (Luck et al. 2009). We therefore focus on the ecosystem processes that underlie wetland ES potential and the effects of vegetation on these processes.

Vegetation, usually the largest biotic component of an ecosystem, influences physical ecosystem structure, energy, water, and nutrient cycles. Plant functional traits are increasingly used as key links between species and ecosystem processes (Hooper et al. 2005). There is growing evidence that ecosystem processes are determined by the product of the distribution of the relevant traits and the relative abundance of species in the community (biomass ratio hypothesis, Grime 1998; Díaz et al. 2007), i.e., processes are driven by the community-weighted mean trait (CWMT) value. Such mechanistic frameworks open avenues for predictive modeling of spatial ES distribution and potential change due to species compositional changes (e.g., Lavorel et al. 2011).

While the field has seen rapid progress during the last decade (Lavorel 2013), most of the work has focused on grasslands (De Bello et al. 2010), which differ from other habitats with regard to important processes and services. Wetlands denote a group of diverse habitat types that have in common a water table close to the soil surface and plants adapted to frequently/continuously anaerobic soil conditions (Mitsch and Gosselink 2007). While other characteristics are highly variable, in terms of hydrology, three wetland types are often recognized: bogs, fens, and riparian wetlands. Bogs are rainwater-fed peatlands, extremely nutrient poor and acidic. Fens also accumulate peat and vary in pH and productivity depending on the focal catchment. Riparian wetlands (shoreline vegetation and marshes) can accumulate peat, but are often relatively more productive, with plants adapted to frequent and recurring disturbances such as seasonal flooding. Bryophytes often dominate bogs and certain types of fens, whereas vascular plant biomass is higher in riparian wetlands and some fens (Rydin and Jeglum 2013). Because of these profound differences, ES and relevant processes will also differ between wetland types.

Most fundamental physiological constraints known from terrestrial plants are presumably also valid in wetland species. A few noteworthy studies exist, which focus on trait effects on particular wetland processes (e.g., Engelhardt and Ritchie 2001; Sutton-Grier et al. 2013), but efforts to generalize trait–ES relationships for wetlands remain scarce.

Predicting ecosystem functioning under environmental change via the combination of functional response and effect traits remains elusive in spite of conceptual advances (Suding et al. 2008; Lavorel 2013). Correlative species distribution modeling (SDM) offers an alternative to trait-based prediction when the nature of response traits is uncertain or quantitative data are sparse. SDM links current known species’ occurrences to environmental conditions and predicts occurrence probability under future conditions. By modeling the response of species to multiple climate variables, SDM in a sense estimates an integrated climate response trait. Combining SDM with functional effect traits allows for estimation of future change in the CWMT value of traits relevant to ES. This is attractive because it allows for estimation of the *direction* of potential change in ES due to climate change even in the absence of detailed species abundance data and quantitative estimates of trait–ES linkages.

We here use SDM to predict species compositional change and to provide an estimate of potential changes in mean community traits. We focus on trait-process relationships relevant for wetlands that have been reported in recent literature reviews (Cornwell et al. 2008; De Deyn et al. 2008; De Bello et al. 2010) and for which trait data were available (Table 1). These processes can be linked to the three key wetland ES: flood attenuation, retention and removal of excess nutrients from runoff, and carbon sequestration (CO_2_ and methane) (Zedler 2003; Mitsch and Gosselink 2007). Our analysis focuses on regional species’ compositional changes in bogs, fens, and riparian wetlands in the Norrström Drainage Basin (NDB) in central Sweden, which includes two of Sweden’s largest lakes (Mälaren and Hjälmaren) and drains into the Baltic Sea.

**Table 1:** The functional traits used, assumed relationships to ecosystem processes, and effect of processes on ecosystem services (signs indicate the effect of an increased trait value; from −− to ++; see text for references)

Trait group	Trait	Processes	Effect −−, −, 0, +, ++		
			Flood attenuation	Nutrient retention	C sequestration
Structural	Canopy Height	Surface flow resistance (+)	++		
		Particle retention, sedimentation (+)		++	+
		Canopy interception,Transpiration, Infiltration (+)	+		
		Standing biomass (+)			+
	Clonality form	Surface flow resistance (tussocks +)	++		
		Belowground biomass (+)	+	+	+
		Nutrient storage (rhizomatous +)		+	
Leaf	Leaf Persistence	Soil oxygenation in winter (evergreen +)		+	
		Decomposability (evergreen −)		+	+
	SLA	RGR (+)	+	++	−
		NPP (+)	+	++	+
		Litter amount (+)	+	+	+
		Decomposability (+)		−	−
Root	Root depth	Soil oxygenation (+)		+	
		Soil stability (+)	+		+
		Belowground biomass (+)			+
	Mycorrhiza	Nutrient uptake rate (+)		+	
		Standing biomass (+)			+
		Soil respiration (+)		–	–
Hydrophytes	Body Flexibility	Flow resistance (−)	−		
	Space occupancy	Flow resistance (+)	++		
		Particle retention (+)		+	+
		Nutrient uptake rate (+)		+	−

## Materials and methods

### Species

**Fig. 1:**
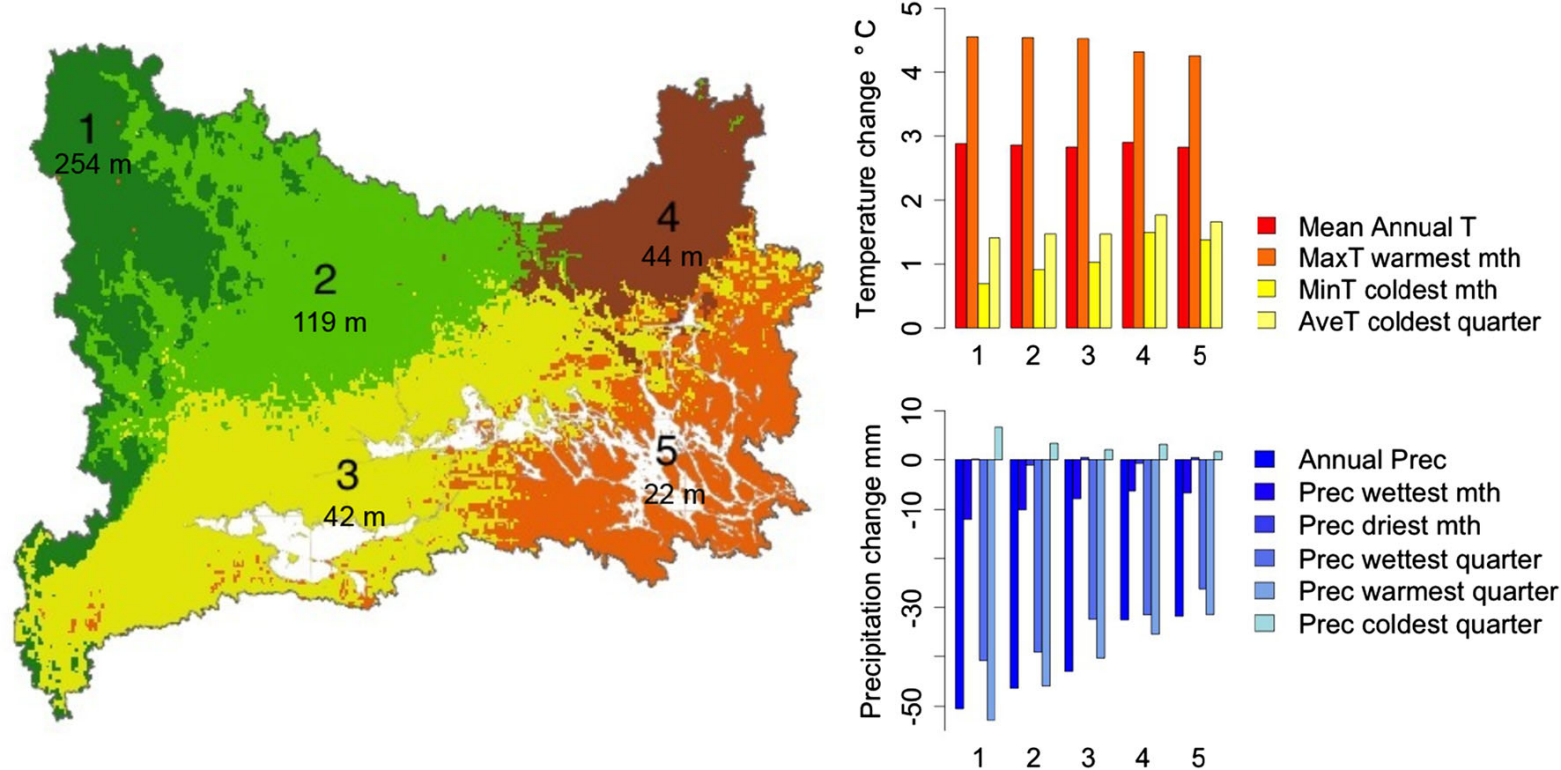
The five regions of species change in the NDB (region number and average elevation) and predicted temperature and precipitation changes. Mean annual temperature increases slightly more in the west, while the colder season gets relatively warmer in the east. Precipitation changes show a distinct gradient with strongest decreases in the west except during the coldest quarter, where precipitation slightly increases

Species’ occurrence data for the whole of Sweden were compiled from the Swedish National Wetland Inventory (Våtmarksinventeringen (VMI), Gunnarsson and Löfroth 2009), a nationwide effort where >4000 wetlands were surveyed in the field, generating plant species lists with estimates of plant abundance (on a scale of 1–3, details in Electronic Supplementary Material). We selected 113 functionally important wetland plants that are common and abundant in any of the three wetland classes with the goal of covering those species that together can dominate community biomass (Table 2). To address spatial heterogeneity within the catchment, we split the NDB into five regions of similar community change based on nonhierarchical K-means clustering of predicted species changes (Fig. 1).

**Table 2:** List of species used for each wetland class (bogs (30 species), fens (45 species), and riparian wetlands (38 species; for 17 hydrophyte species see Electronic Supplementary Material, Table S4). Species that are common in more than one wetland class may appear twice. Plant functional groups (PFGs) are subjective and broadly based on main life form, height, and woodiness, with the exception of *Equisetum* spp. which are placed in a separate, more taxonomically based group due to their unique characteristics. As most grass species in our study are able to develop aerenchyma, we abstained from separating grasses and sedges. For calculation of community-weighted mean traits (CWMT), pteridophytes, graminoid, and herbaceous plants are combined into a single field layer

PFG	Bog	Fen	Riparian
Moss	*Pleurozium schreberi*	*Calliergonella cuspidata*	*Sphagnum riparium*
	*Sphagnum angustifolium*	*Campylium stellatum*	*Sphagnum squarrosum*
	*Sphagnum balticum*	*Scorpidium cossonii*	
	*Sphagnum capillifolium*	*Scorpidium revolvens*	
	*Sphagnum cuspidatum*	*Scorpidium scorpioides*	
	*Sphagnum fuscum*	*Sphagnum centrale*	
	*Sphagnum magellanicum*	*Sphagnum compactum*	
	*Sphagnum majus*	*Sphagnum fallax*	
	*Sphagnum rubellum*	*Sphagnum imbricatum*	
	*Sphagnum russowii*	*Sphagnum lindbergii*	
		*Sphagnum palustre*	
		*Sphagnum papillosum*	
		*Sphagnum pulchrum*	
		*Sphagnum subsecundum*	
		*Sphagnum warnstorfii*	
		*Warnstorfia exannulata*	
Pteridophytes		*Equisetum fluviatile*	*Equisetum fluviatile*
		*Equisetum sylvaticum*	*Equisetum palustre*
			*Equisetum sylvaticum*
Graminoid	*Carex limosa*	*Agrostis canina*	*Agrostis stolonifera*
	*Carex magellanica*	*Carex canescens*	*Calamagrostis canescens*
	*Eriophorum vaginatum*	*Carex chordorrhiza*	*Calamagrostis purpurea*
	*Rhynchospora alba*	*Carex lasiocarpa*	*Carex acuta*
	*Scheuchzeria palustris*	*Carex limosa*	*Carex aquatilis*
	*Trichophorum cespitosum*	*Carex magellanica*	*Carex canescens*
		*Carex nigra*	*Carex rostrata*
		*Carex rostrata*	*Carex vesicaria*
		*Carex vesicaria*	*Deschampsia cespitosa*
		*Eriophorum angustifolium*	*Glyceria maxima*
		*Molinia caerulea*	*Juncus effusus*
		*Phalaris arundinacea*	*Juncus filiformis*
		*Phragmites australis*	*Phalaris arundinacea*
		*Schoenus ferrugineus*	*Phragmites australis*
		*Trichophorum cespitosum*	*Scirpus sylvaticus*
			*Typha latifolia*
Herbaceous	*Andromeda polifolia*	*Drosera intermedia*	*Calla palustris*
	*Drosera rotundifolia*	*Menyanthes trifoliata*	*Caltha palustris*
	*Rubus chamaemorus*	*Narthecium ossifragum*	*Cirsium palustre*
		*Potentilla palustris*	*Filipendula ulmaria*
		*Viola palustris*	*Iris pseudacorus*
			*Lycopus europaeus*
			*Lysimachia thyrsiflora*
			*Lysimachia vulgaris*
			*Lythrum salicaria*
			*Potentilla palustris*
Shrub	*Betula nana*	*Betula nana*	*Myrica gale*
	*Calluna vulgaris*	*Calluna vulgaris*	*Salix lapponum*
	*Empetrum hermaphroditum*	*Myrica gale*	*Salix caprea*
	*Empetrum nigrum*	*Salix lapponum*	*Salix cinerea*
	*Erica tetralix*	*Salix repens*	
	*Rhododendron tomentosum*	*Vaccinium oxycoccus*	
	*Vaccinium myrtillus*		
	*Vaccinium oxycoccus*		
	*Vaccinium uliginosum*		
Tree	*Betula pubescens*	*Betula pubescens*	*Alnus glutinosa*
	*Pinus sylvestris*		*Alnus incana*
			*Betula pubescens*
			*Fraxinus excelsior*
			*Picea abies*

### Traits

Trait data for vascular plants (mean value per species) were obtained from public databases, with the majority from the LEDA traitbase (Kleyer et al. 2008; Electronic Supplementary Material, Table S3). Gaps in trait data were filled with estimates based on phylogenetic relationships (generated with phyloGenerator) using PhyloPars (Bruggeman et al. 2009). This approach resulted in complete datasets for the continuous traits: specific leaf area (SLA), canopy height (CH), and root depth (RD) (Table 1). SLA is a good positive correlate of relative growth rate (RGR) and negatively related to leaf lifespan. CH often scales allometrically with other size traits such as aboveground biomass and lateral spread (Cornelissen et al. 2003). Root depth (RD) can be related to the extent of potential rhizosphere oxygenation (affecting denitrification and decomposition) and root biomass (affecting belowground carbon storage and soil stabilization) (De Deyn et al. 2008).

In addition, we used the following categorical traits: Leaf persistence (levels: evergreen, summergreen) relates to leaf decomposability; clonality forms (nonclonal, tussock-forming, and rhizomatous) has implications for plant structure relating to flow attenuation (tussocks, Bouma et al. 2013) and for relatively greater investment in root biomass (rhizomatous, Cronk and Fennessy 2001); the presence of mycorrhizal symbiosis (nonmycorrhizal, facultative, obligatory) facilitates nutrient uptake and plant growth (Hempel et al. 2013). Aquatic plants were modeled as a separate functional group, characterized by the categorical traits: body flexibility (degrees of deformation under flow pressure—rigid, elastic, or soft) and space occupancy (small, medium, or large), both of which relate to water flow resistance (Willby et al. 2000; Electronic Supplementary Material, Table S4).

### Modeling trait change

Species distributions were modeled for the whole of Sweden with Maxent (Philips et al. 2006) using environmental data listed in Table 3 as input. Maxent is the method of choice for presence-only data, performs well with a varying number of occurrence points, and is insensitive to correlated predictors (Philips and Dudík 2008). Only climatic variables varied in the future scenario models; land use change or atmospheric nitrogen deposition could affect species additionally and potentially more strongly (Mitsch and Gosselink 2007; Limpens et al. 2011) but were not included in the scenario of this study due to lack of spatial predictions of future changes (SDM details in Electronic Supplementary Material).

**Table 3:** Environmental predictors included in final SDMs, current average values for NDB, predicted change, and data sources. As future scenario, we used predictions by 2070 of the HadGEM2-AO model at an intermediate emission scenario (RCP 6.0). For the Norrström Drainage Basin (NDB), the model predicts an increase in mean annual temperature of +2.85 °C, and a decrease in annual precipitation of −6.7 %

Predictor	Current mean NDB	Mean change NDB	Region 1	Region 2	Region 3	Region 4	Region 5	Source
Annual mean temperature (°C)	5.60	+2.85	+2.88	+2.86	+2.83	+2.9	+2.83	(1)
Maximum temp of warmest month (°C)	21.58	+4.46	+4.56	+4.55	+4.53	+4.31	+4.25	(1)
Minimum temp of coldest month (°C)	−7.75	+1.07	+ 0.7	+0.92	+1.03	+1.5	+1.39	(1)
Average temp of coldest quarter (°C)	−3.67	+1.54	+1.42	+1.48	+1.48	+1.77	+1.67	(1)
Annual precipitation (mm)	619	−42	−50	−46	−43	−32	−32	(1)
Precipitation of wettest month (mm)	76	−9	−12	−10	−8	−6	−7	(1)
Precipitation of driest month (mm)	31	0	0	−1	0	−1	0	(1)
Precipitation of wettest quarter (mm)	215	−34	−41	−39	−32	−31	−26	(1)
Precipitation of warmest quarter (mm)	203	−41	−53	−46	−40	−35	−31	(1)
Precipitation of coldest quarter (mm)	127	+3	+7	+3	+2	+3	+2	(1)
Elevation (m)	89							(2)
pH	4.64							(3)
Soil types (10 classes)								(4)
Bedrock types (15 classes)								(4)

(1) Hijmans, R.J., S.E. Cameron, J.L. Parra, P.G. Jones, and A. Jarvis. 2005. Very high resolution interpolated climate surfaces for global land areas. International Journal of Climatology 25:1965–1978. Retrieved October 10, 2013, from www.worldclim.org

(2) Swedish National Land Survey (Lantmäteriet). 2010. GSD-Höjddata, Grid 50+.© Lantmäteriet, i2012/899

(3) © Swedish University of Agricultural Sciences (SLU). 2013

(4) © Swedish Geological Survey (SGU). Retrieved November 30, 2013, from www.sgu.se/sgu/sv/produkter-tjanster/databaser

#### Modeling relative abundance and maximum potential biomass

Our goal was to estimate potential biomass density from the abundance data of the VMI inventory. The presence points were split into three datasets—one for each of the three abundance classes (single individuals, frequent, and dominant), and Maxent was run separately on occurrences of each abundance class. For the modeled occurrence probabilities of the three abundance classes, a weighted average was calculated as approximation of a prediction of relative abundance (weights: single = 0.01, frequent = 0.1, dominant = 1). While simple occurrence data could already provide reasonable estimates of abundance (Van Couwenberghe et al. 2013), this method allowed us to model an occurrence value that is more closely related to estimated field abundance than the usual output of Maxent. Yet we treat the results as a basis for discussion rather than a solid prediction.

The mass ratio hypothesis poses that the community mean trait value weighted for relative biomass (CWMT) is the strongest driver of ecosystem processes (Grime 1998). The relationship between the estimate of abundance from the above SDM approach and the potential relative biomass of each species in the community can be inferred by means of general allometric scaling relationships between species height, individual biomass, and density. Specifically, height is proportional to biomass *M* ^0.264^ (Niklas and Enquist 2001), and maximum abundance *N* scales with *M* ^−0.757^ (Belgrano et al. 2002). Putting these relationships together (Electronic Supplementary Material), we estimated maximum potential biomass (kg dry weight m^−2^) from height (m) as *B*_max_ = 1.769*e*05*(*H*/*b*)^(−0.757/*a*)^ (where *a* = 0.264, *b* = 2.58; for trees: *a* = 0.345, *b* = 3.71).

#### CWMT change

Based on this potential relative biomass, we arrived at first guiding estimates of potential changes in relevant CWMT values in bogs, fens, and riparian wetland communities across the NDB. Changes in CWMT values were calculated separately for the field and shrub layers within each wetland type, for each 500-m cell across the NDB, and averaged within the five identified regions of community change.

Note that relative biomass in each cell represents proportions of species according to modeled environmental suitability; translation to local communities (habitat filter and competitive effects) is beyond the scope of this study. The relative contribution of each species to CWMT changes was assessed via the standardized mean squared error resulting from excluding that species from calculations (Electronic Supplementary Material, Table S5). Since trees form a very different functional group than field or shrub layer species and due to their low species number, we treat them separately. As we had neither trait data nor size estimates of bryophytes, effects of distributional changes of bryophytes (in our case, only mosses) are discussed based on estimated relative abundance and compositional shifts only.

## Results

The general direction of CMWT changes is overall consistent across the whole NDB (with the exception of bog and riparian wetland shrub layers), albeit differing in strength. Regional differences in the riparian shrub layer are mostly driven by an inverse pattern of change of *Salix cinerea* and *S. caprea* and, in the bog shrub layer, by regional differences in the increase of relative biomass of *Rhododendron tomentosum*. Changes in individual species’ relative biomass are reported in Electronic Supplementary Material, Fig. S2).

### Climate change-driven trait shifts

#### Continuous traits: CWMT change

**Fig. 2:**
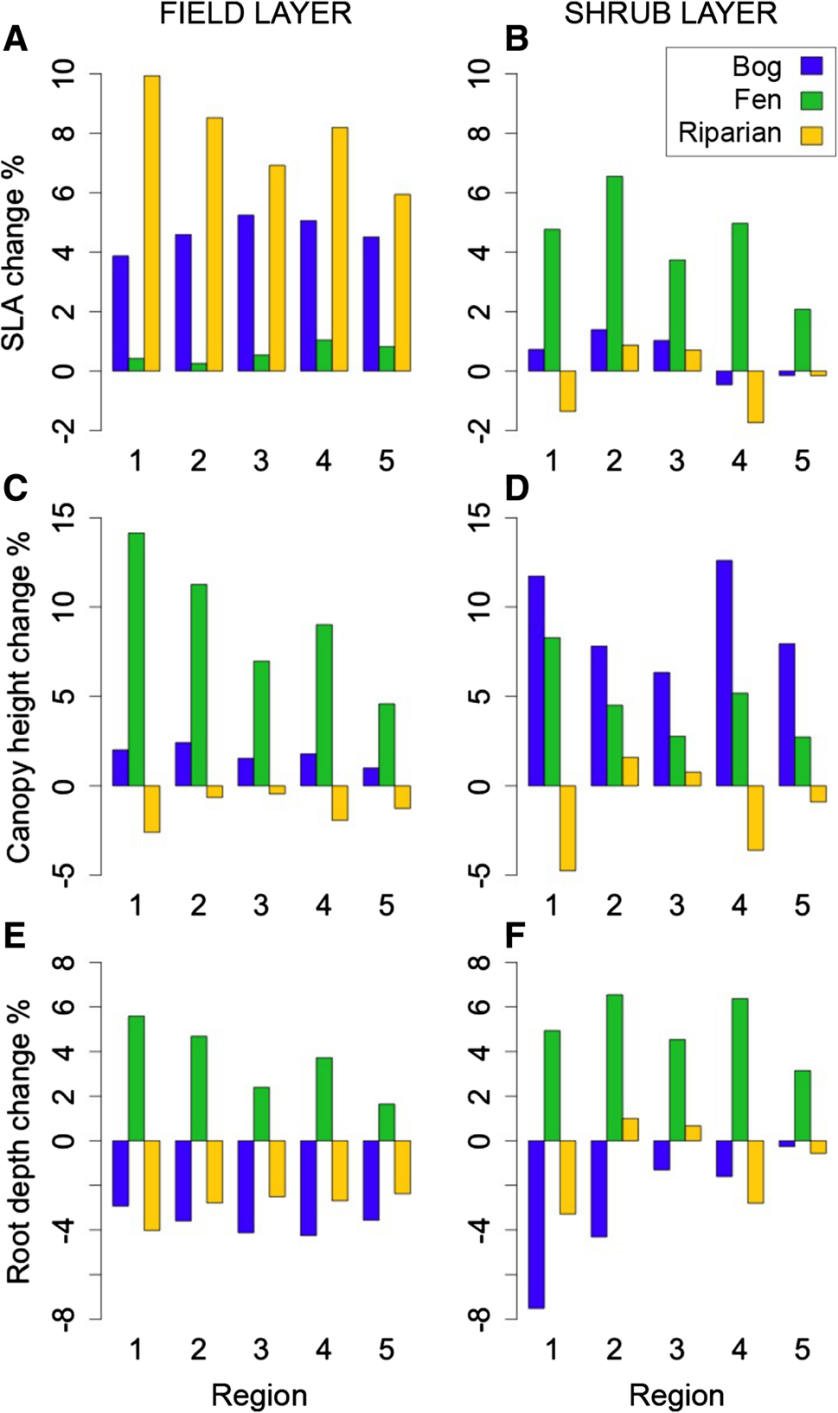
Predicted CWMT change for the three wetland types in the five regions. The *left-hand side* shows changes in the field layer, the *right-hand side* shows changes in the shrub layer of specific leaf area (SLA; **a**, **b**), canopy height (CH; **c**, **d**), and root depth (RD; **e**, **f**)

SLA increases in all wetland types, most strongly in the riparian field layer, and in the field layer of bogs (Fig. 2a, absolute CWMT values in Electronic Supplementary Material, Table S6), suggesting a trend to higher net primary production (NPP, due to increased relative growth rates) and faster nutrient turnover (higher nutrient uptake and decomposition rates). In the fen field layer, differences are minor, whereas among shrub layers, SLA is predicted to increase most in fens, with only small changes in bogs and riparian wetlands (Fig. 2b).

In the field layer, CH change is minor in bogs and riparian wetlands, but pronounced in fens, particularly so in regions 1, 2 (Fig. 2c), suggesting larger standing biomass, higher potential canopy interception and transpiration rates, as well as increased flow resistance. CH increases in the shrub layer of bogs and fens, whereas riparian shrub CH shows regionally varied responses (Fig. 2d).

**Fig. 3:**
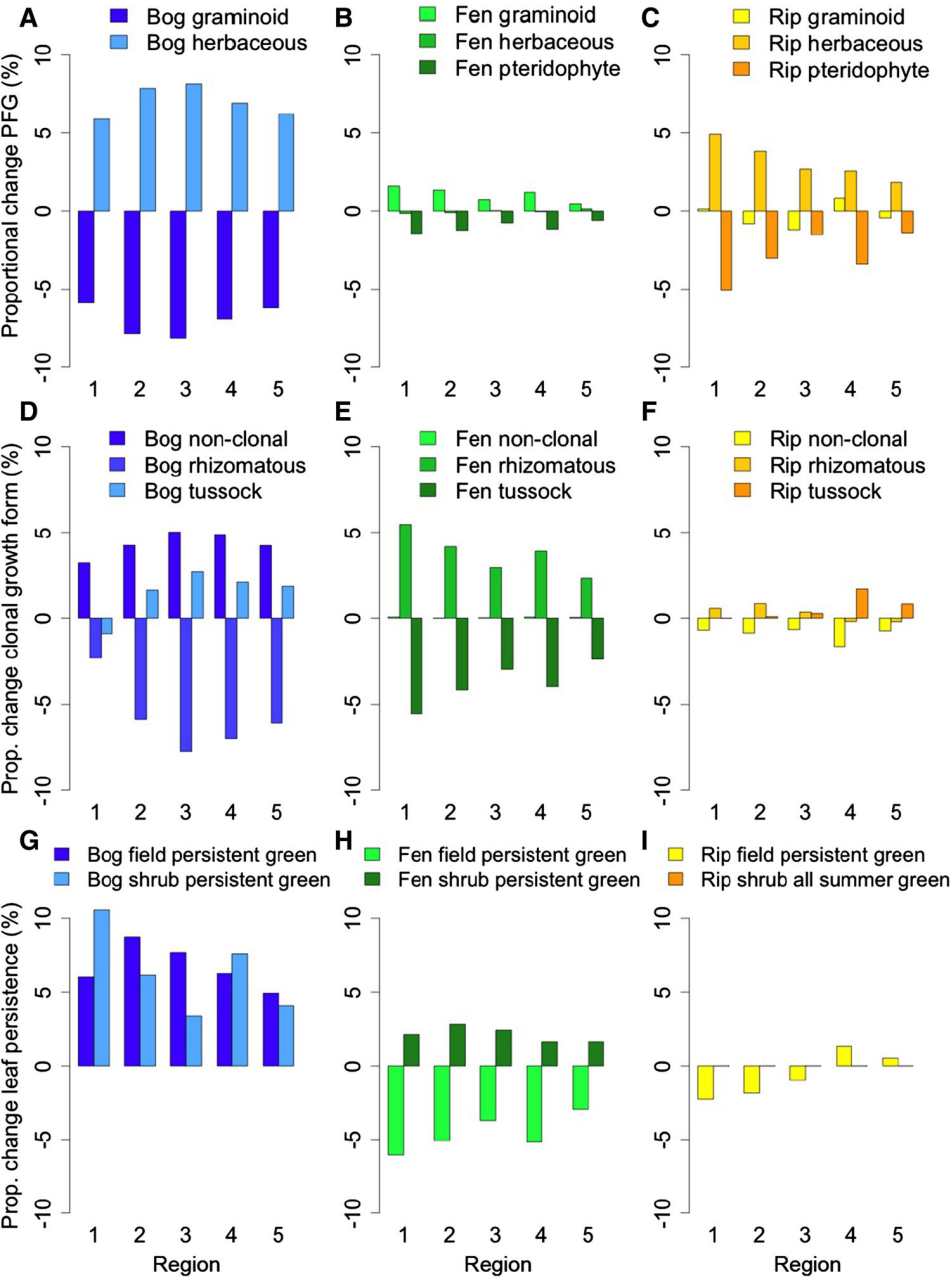
Predicted proportional change of categorical trait levels in the three wetland types, for the five regions. **a**–**c** Plant functional groups (PFGs) in the field layer. **d**–**f** Clonal growth form in the field layer. **g**–**i** Leaf persistence in both field and shrub layers, shown as change in proportion of persistent green species

The RD decreases in field layers of bogs and riparian wetlands, whereas in fens, RD is predicted to increase in the field layer (Fig. 2e), consistent with the pattern of change in rhizomatous species (Fig. 3d, e), suggesting larger belowground biomass in fens. Also in the shrub layer, RD increases in fens (Fig. 2f). In shrub layers of bogs and riparian wetlands, RD overall tends to decrease, but shows a spatially more variable pattern (Fig. 2f), again due to above-mentioned regional differences in relative changes of *R. tomentosum*, *S. cinerea* ,and *S. caprea*.

#### Categorical traits: Proportional change

**Fig. 4:**
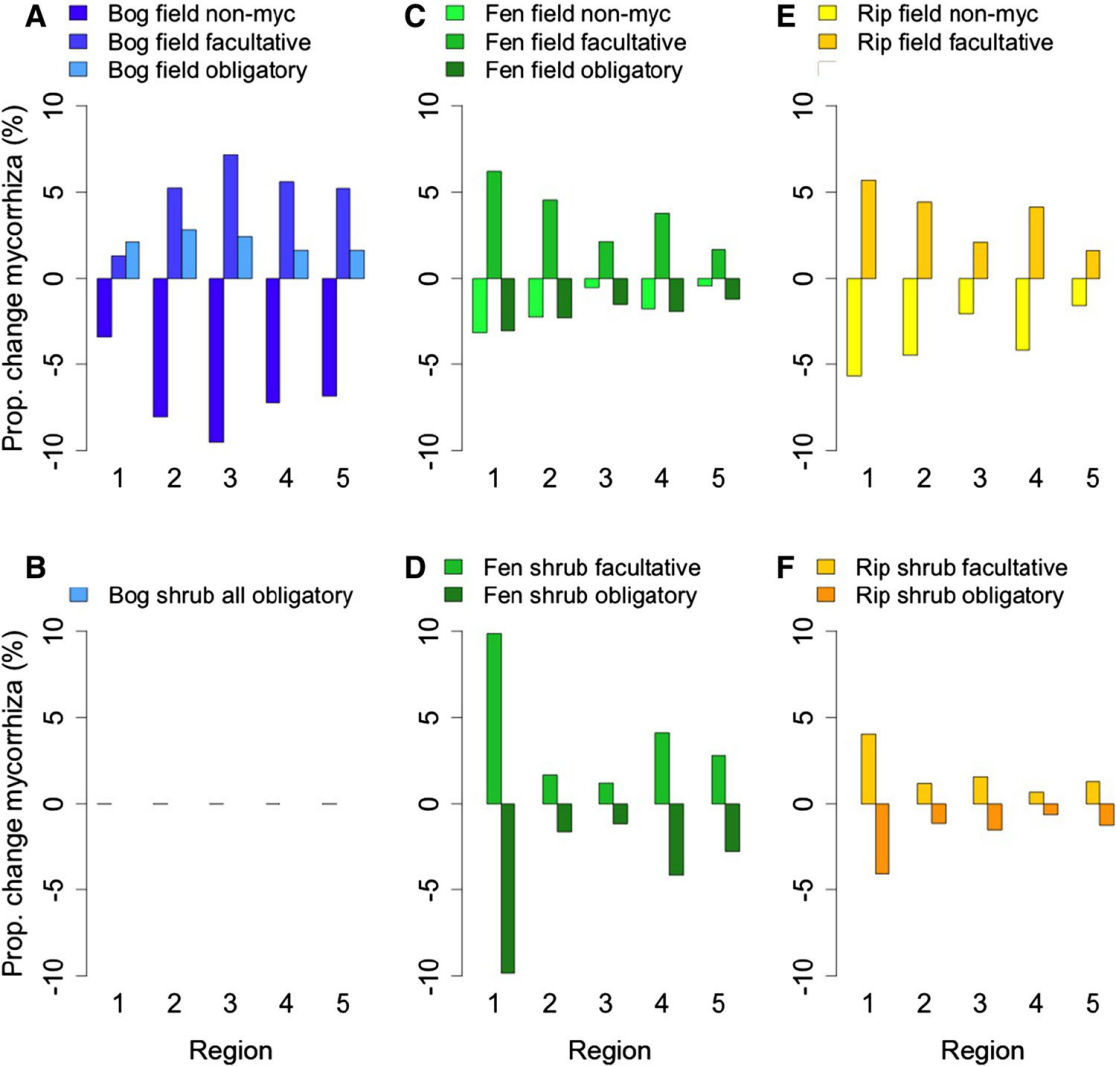
Predicted proportional change of mycorrhizal association, shown separately for the field layer (**a**, **c**, **e**) and shrub layer (**b**, **d**, **f**) in the three wetland types. There is no change in the bog shrub layer as all species are obligatory mycorrhizal

Shifts to higher proportions of herbaceous plants in bogs and riparian wetlands (Fig. 3a, c), and to more summer green species in the fen field layer (Fig. 3h), mirror increases in SLA. The decrease of rhizomatous species in bogs (Fig. 3d) might decrease nutrient storage in belowground biomass but could slow peat decomposition via reduced soil oxygenation, while increases in tussock-forming species would benefit water flow resistance; in fens, the opposite pattern emerges (Fig. 3e). Increases in persistent green species in bogs and the fen shrub layer (Fig. 3g, h) suggest more recalcitrant litter with lower decomposition rates, slightly counteracting SLA increases. The shift from nonmycorrhizal to more facultative mycorrhizal species in all field layers (Fig. 4a–d) indicates higher potential for nutrient uptake and increased plant productivity, but this might be counteracted by decreases in obligatory mycorrhizal species among shrubs in fens and riparian wetlands (Fig. 4d, f).

**Fig. 5:**
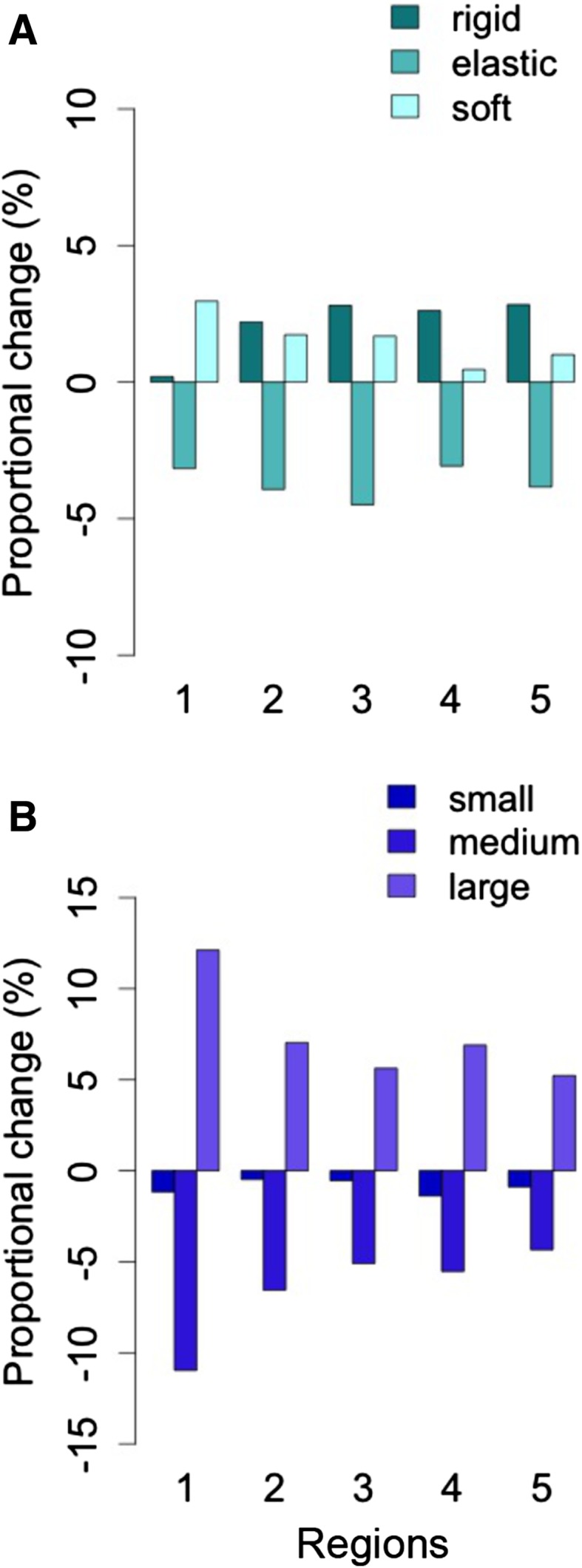
Proportional change in body flexibility (**a**) and space occupancy (**b**) among hydrophytes for the five regions

Among hydrophytes, more lifeforms with rigid structure become dominant at the expense of mainly elastic lifeforms (Fig. 5a), except in region 1. In terms of space occupancy, a clear shift toward larger species is observed (Fig. 5b), positively affecting particle retention. Both trends indicate higher potential water flow resistance.

#### Bryophyte distributional change

Among bog species, the wet hollow species *Sphagnum majus* and *S. cuspidatum* and the hummock species *S. rubellum* are predicted to decrease, mostly in the western NDB (Electronic Supplementary Material, Fig. S3). This might decrease the productivity of bogs since hollow species are suggested to be more productive than hummock species (Gunnarson 2005). On the other hand, only small changes are predicted for other dominant species in the NDB (e.g., the hummock species *S. fuscum* and *S. capillifolium*). In fens, *Calliergonella cuspidata* and *Sphagnum fallax* are predicted to increase noticeably throughout NDB; both species are also favoured by increased nutrient inputs (not modeled), thus potential increases in nitrogen deposition might further emphasize this change (Limpens et al. 2011). *Scorpidium revolvens* (rich fens) and *Sphagnum pulchrum* (poor fens) decline, both fen species typical of wetter parts. Also *Sphagnum imbricatum* declines.

#### Trees

Maximum potential biomass is predicted to increase for most tree species (*Pinus sylvestris, Betula pubescens, Fraxinus excelsior*, and *Alnus glutinosa*). Only *Alnus incana* and *Picea abies* are predicted to decrease. Due to long generation times and uncertainties in estimating realized biomass for trees, we here restrict the discussion to one particular species that might serve as an example: *A. glutinosa* increases strongly across the NDB, overcompensating the northward shift of *Alnus incana*. It features key effect traits (nitrogen fixation symbionts, deep soil oxygenation, high inundation tolerance, and flow resistance) with implications for nutrient retention and flood attenuation.

### Potential implications of trait change for ES

#### Flood attenuation

Water flow regulation is an important aspect of wetland functioning and benefits humans not only by attenuating the devastating effects of floods, but also by filtering particles and aiding sedimentation (contributing to *nutrient retention*) as well as maintaining soil water saturation (reducing decomposition rates and aiding in *C sequestration*) (Zedler 2003). The largest effect of vegetation on water flow regulation in riparian wetlands is via physical resistance, slowing down of surface waters, and temporary storage. Among hydrophytes, the shift toward larger and more rigid species indicates an increased potential for flow resistance and the attenuation of floodwater peaks within streams (Nepf 2012). In riparian wetlands, a relative increase of tussock-forming graminoids, along with the ubiquitous increase of *A. glutinosa* points to an increased potential of vegetation to slow down overflowing river waters and decrease flood damage (Claessens et al. 2010; Bouma et al. 2013). Increased CH in fens and bogs could theoretically cause longer water-retention times, higher canopy interception, infiltration and transpiration rates, and thus a generally stronger role in the hydrological cycle. Given these relationships, the flood attenuation service would benefit from the predicted changes. In fens and bogs, however, the species-specific waterholding capacity of bryophytes, for which data are insufficient, is important for flow regulation (Rydin and Jeglum 2013). A shift from *Sphagnum* dominated to *Calliergonella* dominated fens might decrease the waterholding capacity and hence increase downstream flood risk.

#### Nutrient retention

Major plant effects on the retention and removal of nutrients are via mediation of plant uptake and storage, denitrification (direct loss of N_2_ to the atmosphere), and facilitation of sedimentation (Saunders and Kalff 2001). Mycorrhizal symbiosis generally increases nutrient uptake rates and can increase plant productivity and standing biomass (Hempel et al. 2013). The overall shift toward higher proportions of mycorrhizal species in all field layers might thus lead to higher standing biomass and potentially nutrient storage, even if most species are facultatively mycorrhizal. Mycorrhizal infection in facultative species is known to decrease with higher soil water saturation (Muthukumar et al. 2004), such that general decreases in precipitation could be positive for symbioses. Among shrubs, however, obligatory mycorrhizal species decrease, so the overall effect of proportional shifts in mycorrhizal types is inconclusive. The observed trend for increases in SLA indicates an increase of fast-growing species, higher community NPP and larger above-ground biomass and litter amount, i.e., overall increased nutrient uptake. On the other hand, the usually higher leaf nutrient content of high-SLA species implies more easily decomposable litter, faster decomposition rates, and reduced storage times (Cornwell et al. 2008).

Denitrification increases under input of more and higher quality litter, i.e., more and better usable carbon sources for denitrifying microbes (Sutton-Grier et al. 2013). Denitrification itself depends on anoxic conditions, but its rates are the highest at the interface between anoxic and oxygenated layers, where denitrification is coupled to nitrification most efficiently (Mitsch and Gosselink 2007). Plants that are able to oxygenate the rhizosphere around their roots should thus be overall beneficial to denitrification. Assuming that RD is proportional to the volume of soil a plant can oxygenate, higher observed RDs and proportion of rhizomatous species in fens (but not bogs or riparian wetlands) would increase denitrification rates. Better indicators for plant soil oxygenation potential would be root porosity or aerenchymatous tissue in plant species. Most sedges, rushes, and particular functional keystone species like *Phragmites* are characterized by large internal gas-spaces, which allow for diffusion of gases through the plant and oxygenation of the rhizosphere. The observed decrease of relative biomass of some sedges might reduce this effect, but other large species known to contribute substantially to soil oxygenation increase (*Phragmites australis*, *Typha latifolia*) (Jackson and Armstrong 1999). Denitrification rates as well as nutrient-uptake rates (but not long-term storage) might thus overall increase, and therewith the removal of nitrogen from downstream waters.

#### Carbon sequestration

Vascular plants influence C storage via NPP (potentially increasing with larger SLA and CH), the net ecosystem carbon exchange rate (likely to increase with higher NPP and decomposition rates) and peat properties (Limpens et al. 2008). Increased decomposition due to higher-quality litter (as well as higher temperatures) is likely to have a stronger negative effect on C storage than on nutrient retention. Increased proportion of rhizomatous growth forms and RD in fens could be positive for C storage if coincident with greater belowground biomass (Cronk and Fennessy 2001), or negative via higher soil/peat oxygenation that could fuel decomposition. The formation and composition of peat, the major long-term C reservoir in wetlands, is dominated by bryophytes, especially *Sphagna* (Schaepman-Strub et al. 2008) due to their effects on the physicochemical environment (maintenance of high water table, low oxygen availability to decomposers, low temperature, and acidification) and low litter decomposability (De Deyn et al. 2008). Predicted decreases in precipitation, potentially leading to lower water tables (and higher rates of heterotrophic respiration), along with concurrent effects on mosses are here therefore of greater concern than shifts in traits of vascular plants. Out of 28 moss species, only three were predicted to increase substantially in abundance. The increase of the two nutrient-tolerant fen species *C. cuspidata* and *Sphagnum fallax*, and of the relatively less densely growing *S. squarrosum,* could mean a shift to less recalcitrant peat. Long-term C sequestration might thus decrease, even if short-term CO_2_ uptake and C storage increase.

Wetlands are also a significant source of the potent greenhouse gas methane (Mitra et al. 2005). Methanogenesis is dependent on anaerobic conditions, labile litter providing accessible C substrate for methanogens, and the export pathway (Mitsch and Gosselink 2007). Decreasing precipitation and warming should generally decrease water table levels, resulting in a deeper aerobic layer, which would reduce methanogenesis but increase CO_2_ release from heterotrophic respiration. More easily decomposable litter, as produced by plants with higher SLA and faster growth rates, could fuel methanogenesis in the same way as denitrification but will have little effect if it gets decomposed before reaching the anaerobic layer. The most important effect of vascular plants on methane emissions is by providing an export pathway through aerenchymatous plant tissue, whereby methane bypasses the aerobic soil layer where it could be oxidized. Sedge cover has been found to be a strong predictor of methane efflux via plants, but other aerenchymatous plants (e.g., *Juncus* spp.) and plants with open gas flow-through systems (*Phragmites, Typha*) have the same effect (Vasander and Kettunen 2006). As the majority of our study species are able to develop aerenchyma, and as relative increases in some plants that can transport methane are balanced by decreases in others, this effect may not change much. Lower water tables would result in overall lower methane production.

Estimation of the overall effect of vegetation changes on C storage and greenhouse gas emissions is difficult, depends on the timescale considered, and might be more strongly influenced by abiotic factors (nitrogen deposition, climate; Limpens et al. 2008) and land use change (especially drainage). In the case of the scenario of this study, both CO_2_ uptake and release are likely to increase in the short term, whereas methane emission most likely will decrease.

## Discussion

We have proposed an approach to estimate climate change effects on wetland services based on underlying mechanistic links to traits and processes. Within communities, the general trend is a proportional shift toward faster growing, more productive, and taller species, consistent with predictions for climate change-induced trait changes in alpine communities (Dubuis et al. 2013). Our approach does not cover the spread of new or invasive species but models shifts in relative biomass of species that make up communities today. Hence, the overall appearance of communities may not change dramatically, yet CWMT values change markedly, and effects on ecosystem functioning should be noticeable. Our results suggest a potential increase in flood attenuation services, a potential increase in short (but not long)-term nutrient retention, and ambiguous outcomes for carbon sequestration.

**Fig. 6:**
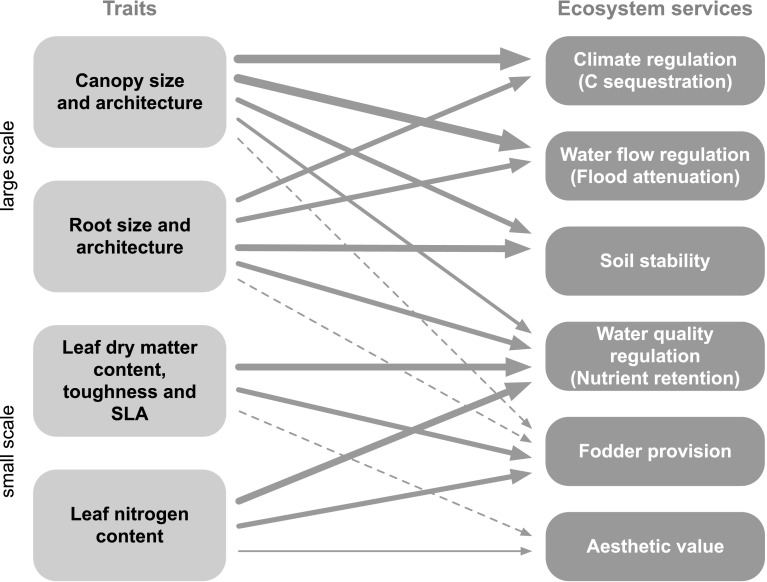
The involvement of plant functional traits in multiple ecosystem service delivery in wetlands (adapted from De Bello et al. 2010). *Larger arrow* thickness for a given trait service relationships indicates proposed stronger relationships based on the literature review of De Bello et al. (2010). *SLA* specific leaf area. Aesthetic value includes multiple cultural services

Plant effects on any ecosystem process are often determined by multiple traits, and most traits affect multiple processes (Eviner and Chapin 2003; Table 1; Fig. 6). Furthermore, the attempt to pin down ecosystem processes that underlie a particular service makes clear that most “services” in fact are service complexes (De Bello et al. 2010; Queiroz et al. 2015), characterized by different aspects that can tradeoff among each other and that differ between wetland types.

The challenges are (1) to identify the suite of traits that affect a process and determine covariation of these traits both within and across species, (2) to disentangle interactive (conflicting or synergistic) effects of traits on different ecosystem processes within wetland types, and (3) to test sensitivities of ES models to CWMTs.

Current efforts to use species community data to predict ES and even change in ES will help to prioritize the set of traits most needed to complement the existing trait data and which presently available traits are most useful for establishing community abundance—ES relationships. For wetlands, these include in no particular order: soil oxygenation capacity, i.e., aerenchyma or root radial oxygen loss, stoichiometry/secondary metabolites, and stem flow properties such as elasticity and Reynolds numbers of single stems and stands (Nepf 2012). In addition, an openly available dataset of traits relating to waterholding capacity and carbon cycling for *Sphagnum* is urgently needed.

Our method aims at translation of predicted changes in species to a shift in important community traits. To arrive at CWMTs, we made the following assumptions: (1) The presence probability can be related to potential abundance. This is plausible albeit not exact (Pearce and Ferrier 2001; Boulangeat et al. 2012; Van Couwenberghe et al. 2013). (2) Biomass density of species locally scales approximately with CH (as proxy for body size). While this is true in general (*R* ^2^ often close to 0.9 over large span of body sizes, Niklas and Enquist 2001), there is large variability within smaller ranges in body size. Field data on species’ relative and maximum realized biomasses are probably the one factor that could increase the accuracy of CWMT predictions dramatically.

We use SDM to predict habitat suitability both currently and in future scenarios. This provides an estimate of the fundamental niche based on regional environmental drivers, not the realized niche subject to establishment and competition filters. To understand both the fundamental niche at the local level as well as the realized niche once species interactions have played out, a multiscale model approach is needed (Boulangeat et al. 2012). Further, dispersal limitation, potential lags (particularly for long lived species), and transient dynamics in distributional shifts are not included. Full realization of predicted community changes is therefore unlikely by 2070, although intrusion of new species might accelerate community trait shifts. While plants can undergo rapid range shifts under climate change (Kelly and Goulden 2008), landscape configuration, wetland connectivity, and the presence of dispersal vectors play important roles in determining the potential for and speed of distributional change (Ozinga et al. 2009). Traits relating to dispersal, establishment, and competitive ability (Weiher et al. 1999) could potentially be used to model these dynamics, an interesting avenue for future research.

Furthermore, we here omitted scenarios for nitrogen deposition, land use change, and exploitation of wetland areas, which could affect ES delivery far more strongly than climate change alone. Conversion of wetlands for other purposes directly removes ES potential and can even cause “disservices,” as in the case of drainage, where peat gets exposed to rapid decomposition and release of carbon accumulated over long time spans (Limpens et al. 2008). Land use patterns in surrounding areas can also indirectly alter ES potential via effects on wetland vegetation. For example, high levels of continuous nutrient loading due to intensive agriculture upstream can decrease local plant diversity and compromise the ability of wetlands to sequester nutrients (Zedler 2003). While land use change can alter wetland services, knowledge of processes and limits of the vegetation’s potential to deliver ES may help in strategic and adaptive planning of where to restore or create wetlands of what type and size in response to land use elsewhere in the catchment (Mitsch and Gosselink 2000).

Climate change effects on ES might appear minor in comparison to direct alteration of the landscape by humans, but they are inevitable and therefore deserve attention. We cannot halt or reverse the changes in climate we have triggered, but knowledge of its implications allows for anticipation of effects and adaptive management.

## Conclusion

The prominence of ES in environmental policy calls for an understanding of the underlying mechanisms. The use of SDM in conjunction with functional traits is a promising research avenue that (1) allows for estimation of the direction of change of ecosystem processes and, potentially, services under climate change, and (2) points to the species that are likely to drive change in functioning due to strong distributional change. Disentangling the balance of multiple ecosystem processes remains a challenge, but for water flow regulation, we found that the involved processes synergistically increased this service. For nutrient retention and carbon sequestration, further data are needed to resolve the species community change-driven net impact on these services. Future research should focus on extension of the knowledge base for relevant traits and processes for a wider range of ecosystems to further operationalize the approach.

## Electronic supplementary material

Below is the link to the electronic supplementary material.

Supplementary material 1 (PDF 20122 kb)

## References

[CR1] Barbier EB (2011). Wetlands as natural assets. Hydrological Sciences Journal.

[CR2] Belgrano A, Allen AP, Enquist BJ, Gillooly JF (2002). Allometric scaling of maximum population density: A common rule for marine phytoplankton and terrestrial plants. Ecology Letters.

[CR3] Boulangeat I, Gravel D, Thuiller W (2012). Accounting for dispersal and biotic interactions to disentangle the drivers of species distributions and their abundances. Ecology Letters.

[CR4] Bouma TJ, Temmerman S, van Duren LA, Martini E, Vandenbruwaene W, Callaghan DP, Balke T, Biermans G (2013). Organism traits determine the strength of scale-dependent bio-geomorphic feedbacks: A flume study on three intertidal plant species. Geomorphology.

[CR5] Bruggeman J, Heringa J, Brandt BW (2009). PhyloPars: Estimation of missing parameter values using phylogeny. Nucleic Acids Research.

[CR6] Claessens H, Oosterbaan A, Savill P, Rondeux J (2010). A review of the characteristics of black alder (*Alnus glutinosa* (L.) Gaertn.) and their implications for silvicultural practices. Forestry.

[CR7] Cornelissen J, Lavorel S, Garnier E, Diaz S, Buchmann N, Gurvich ND, Reich PB, ter Steege H (2003). A handbook of protocols for standardised and easy measurement of plant functional traits worldwide. Australian Journal of Botany.

[CR8] Cornwell WK, Cornelissen JHC, Amatangelo K, Dorrepaal E, Eviner VT, Godoy O, Hobbie SE, Hoorens B (2008). Plant species traits are the predominant control on litter decomposition rates within biomes worldwide. Ecology Letters.

[CR9] Cronk JK, Fennessy MS (2001). Wetland plants: Biology and ecology.

[CR10] De Bello F, Lavorel S, Díaz S, Harrington R, Cornelissen JHC, Bardgett RD, Berg MP, Cipriotti P (2010). Toward an assessment of multiple ecosystem processes and services via functional traits. Biodiversity and Conservation.

[CR11] De Deyn GB, Cornelissen JHC, Bardgett RD (2008). Plant functional traits and soil carbon sequestration in contrasting biomes. Ecology Letters.

[CR12] Díaz S, Lavorel S, De Bello F, Quétier F, Grigulis K, Robson TM (2007). Incorporating plant functional diversity effects in ecosystem service assessments. Proceedings of the National Academy of Sciences of the United States of America.

[CR13] Dubuis A, Rossier L, Pottier J, Pellissier L, Vittoz P, Guisan A (2013). Predicting current and future spatial community patterns of plant functional traits. Ecography.

[CR14] Engelhardt KA, Ritchie ME (2001). Effects of macrophyte species richness on wetland ecosystem functioning and services. Nature.

[CR15] Eviner VT, Chapin III FS (2003). Functional matrix: A conceptual framework for predicting multiple plant effects on ecosystem processes. Annual Review of Ecology Evolution and Systematics.

[CR16] Grime JP (1998). Benefits of plant diversity to ecosystems: Immediate, filter and founder effects. Journal of Ecology.

[CR17] Gunnarsson U (2005). Global patterns of *Sphagnum* productivity. Journal of Bryology.

[CR18] Gunnarsson, U., and M. Löfroth. 2009. The Swedish National Wetland Inventory. Stockholm SEPA: Rapport 5925. (In Swedish).

[CR19] Hempel S, Götzenberger L, Kühn I, Michalski SG, Rillig MC, Zobel M, Moora M (2013). Mycorrhizas in the Central European Flora: Relationships with plant life history traits and ecology. Ecology.

[CR20] Hooper D, Chapin FS, Ewel JJ, Hector A, Inchausti P, Lavorel S, Lawton JH, Lodge DM (2005). Effects of biodiversity on ecosystem functioning: A consensus of current knowledge. Ecological Monographs.

[CR21] Jackson MB, Armstrong W (1999). Formation of aerenchyma and the processes of plant ventilation in relation to soil flooding and submergence. Plant Biology.

[CR50] Kelly, A.E., and M.L. Goulden. 2008. Rapid shifts in plant distribution with recent climate change. *Proceedings of the National Academy of Sciences of the United States of America* 105: 11823–11826. 10.1073/pnas.0802891105 PMC2575286 18697941

[CR22] Kleyer M, Bekker RM, Knevel IC, Bakker JP, Thompson K, Sonnenschein M, Poschlod P, van Groenendael JM (2008). The LEDA traitbase: A database of life-history traits of the Northwest European flora. Journal of Ecology.

[CR23] Lavorel S (2013). Plant functional effects on ecosystem services. Journal of Ecology.

[CR24] Lavorel S, Grigulis K, Lamarque P, Colace M-P, Garden D, Girel J, Pellet G, Douzet R (2011). Using plant functional traits to understand the landscape distribution of multiple ecosystem services. Journal of Ecology.

[CR25] Limpens J, Berendse F, Blodau C, Canadell J, Freeman C, Holden J, Roulet N, Rydin H (2008). Peatlands and the carbon cycle: From local processes to global implications? A synthesis. Biogeosciences Discussions.

[CR26] Limpens J, Granath G, Gunnarsson U, Aerts R, Bayley S, Bragazza L, Bubier J, Buttler A (2011). Climatic modifiers of the response to nitrogen deposition in peat-forming *Sphagnum mosses*: A meta-analysis. New Phytologist.

[CR27] Luck G, Harrington R, Harrison PA (2009). Quantifying the contribution of organisms to the provision of ecosystem services. BioScience.

[CR49] Mitra, S., R. Wassmann, and P. Vlek. 2005. An appraisal of global wetland area and its organic carbon stock. *Current Science* 88: 25–35.

[CR28] Mitsch WJ, Gosselink JG (2000). The value of wetlands: Importance of scale and landscape setting. Ecological Economics.

[CR29] Mitsch WJ, Gosselink JG (2007). Wetlands.

[CR30] Muthukumar T, Udaiyan K, Shanmughavel P (2004). Mycorrhiza in sedges—An overview. Mycorrhiza.

[CR31] Nepf HM (2012). Flow and transport in regions with aquatic vegetation. Annual Review of Fluid Mechanics.

[CR32] Niklas KJ, Enquist BJ (2001). Invariant scaling relationships for interspecific plant biomass production rates and body size. Proceedings of the National Academy of Sciences of the United States of America.

[CR33] Ozinga WA, Römermann C, Bekker RM, Prinzing A, Tamis WLM, Schaminée JHJ, Hennekens SM, Thompson K (2009). Dispersal failure contributes to plant losses in NW Europe. Ecology Letters.

[CR34] Phillips SJ, Anderson RP, Schapire RE (2006). Maximum entropy modeling of species geographic distributions. Ecological Modelling.

[CR35] Phillips S, Dudík M (2008). Modeling of species distributions with Maxent: New extensions and a comprehensive evaluation. Ecography.

[CR36] Pearce J, Ferrier S (2001). The practical value of modelling relative abundance of species for regional conservation planning: A case study. Biological Conservation.

[CR37] Queiroz, C., M. Meacham, K. Richter, A.V. Norström, E. Andersson, J. Norberg, and G. Peterson. 2015. Mapping bundles of ecosystem services reveals distinct types of multifunctionality within a Swedish landscape. *AMBIO* (Suppl. 1). doi:10.1007/s13280-014-0601-0. 10.1007/s13280-014-0601-0 PMC4289005 25576284

[CR38] Rydin H, Jeglum JK (2013). The biology of peatlands.

[CR39] Saunders DL, Kalff J (2001). Nitrogen retention in wetlands, lakes and rivers. Hydrobiologia.

[CR40] Schaepman-Strub G, Limpens J, Menken M, Bartholomeus HM, Schaepman E (2008). Towards spatial assessment of carbon sequestration in peatlands: Spectroscopy based estimation of fractional cover of three plant functional types. Biogeosciences Discussions.

[CR41] SEPA (2012). Compiled information on ecosystem services.

[CR42] Suding KN, Lavorel S, Chapin FS, Cornelissen JHC, Díaz S, Garnier E, Goldberg D, Hooper D (2008). Scaling environmental change through the community-level: A trait-based response-and-effect framework for plants. Global Change Biology.

[CR43] Sutton-Grier AE, Wright JP, Richardson CJ (2013). Different plant traits affect two pathways of riparian nitrogen removal in a restored freshwater wetland. Plant and Soil.

[CR44] Van Couwenberghe R, Collet C, Pierrat J-C, Verheyen K, Gégout J-C (2013). Can species distribution models be used to describe plant abundance patterns?. Ecography.

[CR45] Vasander H, Kettunen A, Wieder K, Witt DH (2006). Carbon in boreal peatlands. Boreal peatland ecosystems.

[CR46] Weiher E, Werf A, Thompson K, Roderick M, Garnier E, Eriksson O (1999). Challenging Theophrastus: A common core list of plant traits for functional ecology. Journal of Vegetation Science.

[CR47] Willby N, Abernethy VJ, Demars BOL (2000). Attribute-based classification of European hydrophytes and its relationship to habitat utilization. Freshwater Biology.

[CR48] Zedler JB (2003). Wetlands at your service: Reducing impacts of agriculture at the watershed scale. Frontiers in Ecology and the Environment.

