# Supplementary material for: Predicting climate change effects on wetland ecosystem services using species distribution modeling and plant functional traits
Source: Ambio. 2015 Jan 9;44(Suppl 1):113–26. doi: 10.1007/s13280-014-0593-9 (PMC4288999; doi:10.1007/s13280-014-0593-9)
Supplement: Supplementary file 1 — Supplementary material 1 (PDF 20122 kb) [file 13280_2014_593_MOESM1_ESM.pdf]

AMBIO

Electronic Supplementary Material

This supplementary material has not been peer reviewed.

Title: **Predicting climate change effects on wetland ecosystem services using species distribution modeling and plant functional traits**

Authors: Helen Moor, Kristoffer Hylander, Jon Norberg

Moor H., K. Hylander, and J. Norberg (2015)

Electronic Supplementary Material – Data

Properties of the Swedish National Wetland Inventory (VMI) data and details on species distribution modeling (SDM) with MaxEnt

The VMI was a nationwide effort to survey the Swedish wetlands below the alpine region, directed by the Swedish Environmental Protection Agency (Gunnarsson and Löfroth 2009). A total of 35 000 sites were included and mapped using aerial photographs. More than 4 000 wetlands were surveyed in the field, generating plant species lists including estimates of plant abundance (on a scale of 1 to 3, from single individuals to dominant).

Only large wetlands were surveyed (> 10 ha in Southern Sweden, > 50 ha in the North), leaving presumably large numbers of small or ephemeral wetlands uncatalogued. Methodology was standardized, but observer's bias is hard to avoid in an effort involving dozens of researchers. Also, since the main aim was description of vegetation types, not all species lists are complete, as own point field surveys revealed.

Within field surveyed wetlands, sub-objects of differing characteristics (hydrology, vegetation type) were delineated and assigned to more detailed wetland types. As species lists and wetland type classification are associated with these sub-objects, our analyses are based on this level, i.e. on spatial scales smaller than the main wetland objects.

These points are typical weaknesses of large datasets like the VMI. Fortunately, species distribution modeling methods are designed to deal with exactly such data.

MaxEnt

We treated occurrence data as presence only, acknowledging that absence from a species list did not necessarily imply true absence. For such data, MaxEnt is the method of choice (Philips et al. 2006, Elith et al. 2006). The method is robust also to the limitation to relatively few occurrence points throughout a species' range (emission of small wetlands) and potential sampling bias (field inventories of high nature value wetlands) (Elith and Leathwick 2009). MaxEnt does not project occurrences into geographical space, but directly into n-dimensional environmental space, thus overcoming issues of spatial autocorrelation (Elith et al. 2011). To counter potential sampling bias in terms of environmental space, we used all inventoried VMI wetlands as a targeted background (Philips and Dudík 2008).

As we modeled current and future distributions of species across the whole extent of Sweden, regional predictions for the Norrström Drainage Basin (NDB) are expected to be robust to weaknesses in primary data as well as the lack of data for surrounding countries (for species with ranges that extend further south).

Predictor variables were pre-selected both based on ecological relevance and driven by data (i.e. strong contribution to models across all species run with a full predictor set). Models were run with default values for parameters, applying linear and quadratic features only. Clamping was avoided to allow for extrapolation to new combinations of environmental drivers. 5-fold cross-validation was performed and model results averaged.

Climatic variables were obtained from Worldclim at a resolution of 0.5° (~630 m) (Hijmans et al. 2005). For the future scenario we used predictions by 2070 of the HadGEM2-AO model at an intermediate emission scenario (RCP 6.0). For the Norrström Drainage Basin (NDB), the model predicts an increase in mean annual temperature of +2.85°C, and a decrease in precipitation of -6.7%. A digital elevation model (DEM) was constructed from 50m resolution elevation data from the Swedish Land Survey (Lantmäteriet 2010). Soil and bedrock data at 1km resolution were obtained from the Swedish Geological Survey (SGU 2013). pH point measurements (n = 20 733) from the Swedish National Forest Inventory (SLU 2013) were interpolated at a resolution of 50 m using a random forest model, with soil type, bedrock type, elevation and land cover as predictors. All rasters were resampled to a common resolution of 500 m with either mean or mode algorithms using GDAL version 1.10.1.

Scaling relationships

The relationship between the estimate of abundance from the SDM approach and the potential relative biomass of each species in the community can be inferred by using general allometric scaling relationships between species height, individual biomass and density. Specifically, height (m) is proportional to biomass $M^{0.264}$ (M in kg dry weight, Niklas and Enquist 2001), according to the power law

$$H = b * M^a \qquad (1)$$

where a = 0.264, b = 2.58, and for trees: a = 0.345, b = 3.71. Hence, individual biomass (kg) can be estimated from height (m) as

$$M = (H / b)^{1/a} \qquad (2)$$

And maximum abundance N (ind. m^{-2}) scales with $M^{-0.757}$ (M in g, Belgrano et al. 2002) according to

$$N = 948 * M\,[g]^{-0.757} = 948 * (M\,[kg]\,/1000)^{-0.757} = 176932.8 * M\,[kg]^{-0.757} \qquad (3)$$

Putting these relationships together we estimated maximum potential biomass (kg dry weight m^{-2}) from height (m) as

$$B_{max} = M * N = 176932.8 * (H/b)^{(-0.757/a)} \qquad (3)$$

Wetland distribution in the NDB

In terms of SDM, spatial heterogeneity of wetland type distribution and species occurrence points is not problematic. Regarding the potential for ES delivery however, wetland distribution in the landscape matters (Mitsch and Gosselink 2001).

The VMI coverage of NDB wetlands (Fig. A1, A-C) appears to be in general agreement with wetland area recorded in Swedish land cover data (Fig. A1, D). In the VMI data, the distributions of different wetland types follow a similar pattern, except for relatively higher numbers of riparian wetlands in the lowlands surrounding lakes Hjälmaren and Mälaren (Fig. A1, C), consistent with a higher percentage of inland marshes there (recorded as CORINE land cover types, Table A1).

In the NDB, a gradient of increasing human population density and percentage of agricultural area towards south and east (Fig. A1, E) coincides with fewer wetlands in the landscape (Fig. A1, A-D). Striking is the generally high density of wetlands in our region 2, characterized by intermediate elevation (average 119 m)

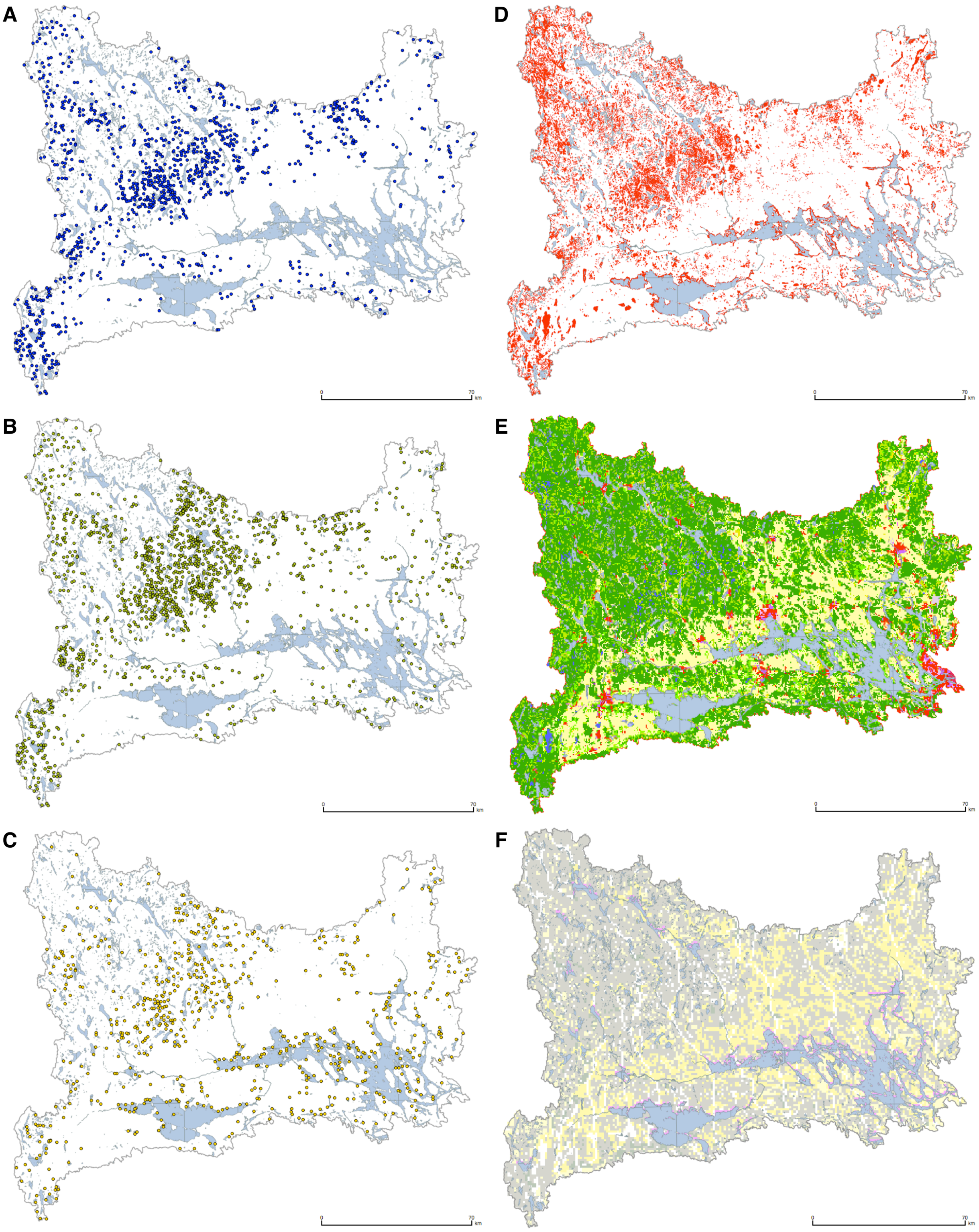

Fig. S1 Regional distribution of wetlands in the NDB. Panels A-C show wetlands catalogued in the VMI: bogs (A, N=1079), Fens (B, N=1391), and riparian wetlands (C, N=697). Panel D shows wetland area extracted from Swedish land cover data (Marktäcke, © Lantmäteriet). Panel E shows CORINE land cover types (EEA), note the concentration of agriculture (yellow) in the lowlands around lakes Hjälmaren and Mälaren, and the increasing degree of urbanization (red) towards the east . Panel F shows soil types (© SGU); clay/silt soils (yellow) are almost absent in region 1. For color legend see tables S1, S2.

and relatively little agriculture. Differences between region 2 and region 1 cannot be explained by human population pressure, with the exception of potentially more intensive forestry towards the west. The very low percentage of clay/silt soils and the higher relief might possibly explain why there are relatively fewer wetlands in region 1 (tables S1, S2; Fig. A1, F). According to CORINE land cover data, however, peat bogs should cover a larger area in region 1 (table S1); hence, regional sampling bias in the VMI data might partially cause this pattern.

To quantitatively estimate the regional distribution of ES potential, the VMI data should preferably be integrated with other, higher resolution datasets. A quantitative link from landscape characteristics to wetland type distribution, in conjunction with local vegetation, could then be used to model the spatial distribution of ES potential. While this was not the focus of the present study, it constitutes a natural extension on the regional scale.

Table S1 Distribution of land cover types across the NDB, as percent cover in each region.

	Land cover type	Region 1	Region 2	Region 3	Region 4	Region 5
	**Urban fabric, industrial, transport, mining**	**0.7**	**3.2**	**6.5**	**4.9**	**14.2**
	**Agricultural**	**0.2**	**12.4**	**39.3**	**27.1**	**25.1**
	Pastures	0.0	0.3	0.7	0.6	1.0
	Forest	82.8	68.2	41.4	57.1	37.2
	Transitional woodland-shrub	11.4	7.9	3.3	9.7	2.2
	Inland marshes	0.0	0.2	0.4	0.5	0.4
	**Peat bogs**	**1.4**	**1.2**	**0.6**	**0.5**	**0.1**
	Water bodies	3.7	8.0	10.4	1.6	25.5

Table S2 Distribution of soil types across the NDB, as percent cover in each region. The small percentage of clay/silt soil in region 1 might contribute to the smaller number of wetlands as compared to region 2.

	Soil class	Region 1	Region 2	Region 3	Region 4	Region 5
	Till, sandy till, clay-till	76.6	61.2	42.4	59.4	41.3
	Esker, sorted sand and gravel	3.2	5.2	5.6	3.6	4.4
	Peat	4.1	3.5	3.1	5.3	1.7
	Sorted sand and gravel	1.5	2.3	3.2	0.7	1.7
	**Clay, silt**	**0.1**	**15.8**	**28.9**	**18.9**	**12.6**
	Bedrock	14.2	9.9	8.4	11.5	16.5

Belgrano, A., A.P. Allen, B.J. Enquist, and J.F. Gillooly. 2002. Allometric scaling of maximum population density: a common rule for marine phytoplankton and terrestrial plants. *Ecology Letters* 5: 611–613.

Elith, J., C.H. Graham, R.P. Anderson, M. Dudík, M., S. Ferrier, A. Guisan, R.J. Hijmans, F. Huettmann, et al. 2006. Novel methods improve prediction of species' distributions from occurrence data. *Ecography* 29: 129–151.

Elith, J., and J.R. Leathwick. 2009. Species Distribution Models: Ecological Explanation and Prediction Across Space and Time. *Annual Review of Ecology, Evolution, and Systematics* 40: 677–697.

Elith, J., S.J. Phillips, T. Hastie, M. Dudík, Y.E. Chee, and C.J. Yates. 2011. A statistical explanation of MaxEnt for ecologists. *Diversity and Distributions* 17: 43–57.

Gunnarsson, U., and M. Löfroth. 2009. Våtmarksinventeringen – resultat från 25 års inventeringar. Nationell slutrapport för våtmarksinventeringen (VMI) i Sverige. Stockholm SEPA: Rapport 5925.

Hijmans, R.J., S.E. Cameron, J.L. Parra, P.G. Jones, and A. Jarvis. 2005. Very high resolution interpolated climate surfaces for global land areas. International Journal of Climatology 25:1965-1978. Retrieved October 10, 2013, from www.worldclim.org

Mitsch, W.J., and J.G. Gosselink. 2000. The value of wetlands: importance of scale and landscape setting. *Ecological*

Economics 35: 25-33.

Niklas, K.J., and B.J. Enquist. 2001. Invariant scaling relationships for interspecific plant biomass production rates and body size. *Proceedings of the National Academy of Sciences of the United States of America* 98: 2922–7.

Phillips, S.J., R.P. Anderson, and R.E. Schapire. 2006. Maximum entropy modeling of species geographic distributions. *Ecological Modelling* 190: 231–259.

Phillips, S., and M. Dudík. 2008. Modeling of species distributions with Maxent: new extensions and a comprehensive evaluation. *Ecography* 31: 161–175.

Swedish National Land Survey (Lantmäteriet). 2010. GSD-Höjddata, Grid 50+.© Lantmäteriet, i2012/899

Swedish University of Agricultural Sciences (SLU). 2013.

Swedish Geological Survey (SGU). Retrieved November 30, 2013, from www.sgu.se/sgu/sv/produkter-tjanster/databaser

---

Trait data for vascular plants

Table S3 Complete set of trait data used for vascular plants (n=69). Asterisks indicate values estimated using PhyloPars (Bruggeman et al. 2008; only root depth, n=40). PhyloPars provides a statistically consistent method of combining empirical observations with a phylogenetic tree to arrive at a complete set of estimates for missing trait values. Since rapid trait divergence can occur even for closely related species, especially when adapting to extreme habitats such as wetlands, we made all efforts to keep the number of estimates obtained via PhyloPars as low as possible. We restricted its use to the estimation of root depth, albeit at high numbers in that case. Root depth values should therefore be viewed with caution.

Species	PFG	SLA ($mm^2 mg^{-1}$)	Canopy height (m)	Root depth (cm)	Leaf persistence	Clonal form	Mycorrhiza
Agrostis canina	graminoid	27.76	0.3	13	summer	Rhizomatous	Facultative
Agrostis stolonifera	graminoid	30.08	0.45	65	persistent	Rhizomatous	Facultative
Alnus glutinosa	tree	16.41	14.33	241	summer	Non-clonal	Obligatory
Alnus incana	tree	31.53	14	117	summer	Non-clonal	Obligatory
Andromeda polifolia	herbaceous	9.31	0.26	76*	persistent	Rhizomatous	Obligatory
Betula nana	shrub	11.72	0.68	133*	summer	Rhizomatous	Obligatory
Betula pubescens	tree	14.39	25	30	summer	Non-clonal	Obligatory
Calamagrostis canescens	graminoid	6.91	1	93*	summer	Rhizomatous	Facultative
Calamagrostis purpurea	graminoid	23.88	1.15	90*	summer	Rhizomatous	Non-myc
Calla palustris	herbaceous	32.82	0.15	39*	summer	NA	Non-myc
Calluna vulgaris	shrub	10.92	0.83	58	persistent	Non-clonal	Obligatory
Caltha palustris	herbaceous	19	0.26	54	summer	Non-clonal	Facultative
Carex acuta	graminoid	15.61	0.9	78*	summer	Rhizomatous	Non-myc
Carex aquatilis	graminoid	14.61	1	80*	persistent	Rhizomatous	Non-myc
Carex canescens	graminoid	28.45	0.32	57*	summer	Rhizomatous	Non-myc
Carex chordorrhiza	graminoid	21.84	0.1	71*	summer	Rhizomatous	Non-myc
Carex lasiocarpa	graminoid	10.44	0.65	67*	persistent	Rhizomatous	Facultative
Carex limosa	graminoid	26.6	0.32	66*	persistent	Rhizomatous	Non-myc
Carex magellanica	graminoid	25.87	0.14	62*	summer	Rhizomatous	Facultative
Carex nigra	graminoid	20.13	0.11	56	summer	Rhizomatous	Facultative
Carex rostrata	graminoid	15.33	0.5	66*	persistent	Rhizomatous	Non-myc
Carex vesicaria	graminoid	18.39	0.55	126	summer	Rhizomatous	Facultative
Cirsium palustre	herbaceous	15.61	0.85	142	persistent	Non-clonal	Facultative
Deschampsia cespitosa	graminoid	11.87	0.43	123	persistent	Tussock	Facultative
Drosera intermedia	herbaceous	28.51	0.04	18*	summer	Non-clonal	Facultative
Drosera rotundifolia	herbaceous	42.92	0.08	22*	summer	Non-clonal	Facultative
Empetrum hermaphroditum	shrub	6.0256	0.2	77*	persistent	Rhizomatous	Obligatory
Empetrum nigrum	shrub	8.4	0.15	77*	persistent	Rhizomatous	Obligatory
Equisetum fluviatile	pteridophyte	9.45	1	107*	summer	Rhizomatous	Non-myc
Equisetum palustre	pteridophyte	8.62	0.3	250	summer	Rhizomatous	Non-myc

Equisetum sylvaticum	pteridophyte	24.75	0.32	141*	summer	Rhizomatous	Facultative
Erica tetralix	shrub	11.82	0.44	79*	persistent	Rhizomatous	Obligatory
Eriophorum angustifolium	graminoid	10.46	0.2	121	persistent	Rhizomatous	Facultative
Eriophorum vaginatum	graminoid	11.99	0.43	61	persistent	Tussock	Facultative
Filipendula ulmaria	herbaceous	14.66	0.79	62	summer	Rhizomatous	Facultative
Glyceria maxima	graminoid	19.79	1.3	83*	summer	Rhizomatous	Facultative
Iris pseudacorus	herbaceous	13.65	0.75	203*	summer	Rhizomatous	Facultative
Juncus effusus	graminoid	7.79	0.9	81	persistent	Tussock	Facultative
Juncus filiformis	graminoid	9.83	0.3	71	persistent	Tussock	Facultative
Lycopus europeus	herbaceous	63.35	0.57	60*	summer	Rhizomatous	Facultative
Lysimachia thyrsiflora	herbaceous	38.09	0.42	50*	summer	Rhizomatous	Facultative
Lysimachia vulgaris	herbaceous	17.3	0.9	89	summer	Rhizomatous	Facultative
Lythrum salicaria	herbaceous	23.13	0.71	64	summer	Non-clonal	Facultative
Menyanthes trifoliata	herbaceous	21.58	0.18	94*	summer	Rhizomatous	Facultative
Molinia caerulea	graminoid	19.72	0.79	96	summer	Tussock	Obligatory
Myrica gale	shrub	11.56	1.22	120*	summer	Rhizomatous	Facultative
Narthecium ossifragum	herbaceous	24.15	0.16	22	persistent	Rhizomatous	Facultative
Phalaris arundinacea	graminoid	20.16	1.45	139	summer	Rhizomatous	Facultative
Phragmites australis	graminoid	13.51	2.5	104*	summer	Rhizomatous	Facultative
Picea abies	tree	17.2	50	103	persistent	Non-clonal	Obligatory
Pinus sylvestris	tree	3.78	34.5	243	persistent	Non-clonal	Obligatory
Potentilla palustris	herbaceous	19.06	0.35	86*	summer	Rhizomatous	Facultative
Rhododendron tomentosum	shrub	10.59	1.02	73*	persistent	NA	Obligatory
Rhynchospora alba	graminoid	16.68	0.25	70*	summer	Rhizomatous	Non-myc
Rubus chamaemorus	herbaceous	11.35	0.1	120*	summer	Rhizomatous	Facultative
Salix caprea	shrub	14.83	6	244	summer	Non-clonal	Obligatory
Salix cinerea	shrub	10.3	2.75	140	summer	Non-clonal	Obligatory
Salix lapponum	shrub	11	0.57	171*	summer	Non-clonal	Obligatory
Salix repens	shrub	20.18	1.05	172*	summer	Rhizomatous	Obligatory
Scheuchzeria palustris	herbaceous	9.78	0.15	80*	summer	Rhizomatous	Non-myc
Schoenus ferrugineus	graminoid	8.69	0.22	70*	persistent	Tussock	Facultative
Scirpus sylvaticus	graminoid	19.02	0.78	170	persistent	Rhizomatous	Non-myc
Trichophorum cespitosum	graminoid	13.19	0.25	78*	summer	Tussock	Facultative
Typha latifolia	graminoid	9.37	1.5	120*	summer	Rhizomatous	Facultative
Vaccinium myrtillus	shrub	24.76	0.31	81*	summer	Rhizomatous	Obligatory
Vaccinium oxycoccos	shrub	13.36	0.39	13*	persistent	Rhizomatous	Obligatory
Vaccinium uliginosum	shrub	14.73	0.29	82*	summer	Rhizomatous	Obligatory
Viola palustris	herbaceous	39.79	0.08	11	persistent	Rhizomatous	Facultative

Primary and secondary sources were

Specific Leaf Area (SLA): LEDA (Kleyer et al .2008);

Canopy Height (CH): LEDA, Grime et al. 2007, PLANTATT (Hill et al. 2004);

Root depth (RD): Kutschera 1960, kindly provided by K. Metselaar (Metselaar et al. 2009);

Leaf persistence: BiolFlor (Klotz et al. 2002);

Clonality form: Ecoflora (Fitter and Peat 1994);

Mycorrhiza: Hempel et al. 2013, Akhmetzhanova et al. 2012.

Akhmetzhanova, A. A., N.A. Soudzilovskaia, V.G. Onipchenko, W.K. Cornwell, V.A. Agafonov, I.A. Selivanov, and J.H.C. Cornelissen. 2012. A rediscovered treasure: mycorrhizal intensity database for 3000 vascular plants species across the former Soviet Union. *Ecology* 93:689.

Hempel, S., L. Götzenberger, I. Kühn, S.G. Michalski, M.C. Rillig, M. Zobel, and M. Moora. 2013. Mycorrhizas in the Central European Flora: relationships with plant life history traits and ecology. *Ecology* 94:1389-1399.

Hill, M.O., C.D. Preston, and D.B. Roy. 2004. *PLANTATT – Attributes of British and Irish Plants: Status, Size, Life History, Geography and Habitats*. NERC. Cambridgeshire: Raven Marketing Group.

Fitter, A.H., and H.J. Peat. 1994, The Ecological Flora Database.Journal of Ecology 82: 415-425. Retrieved from www.ecoflora.co.uk

Grime, J.P., J.G. Hodgson, and R. Hunt. 2007. *Comparative Plant Ecology: a functional approach to common British species.* 2e. Colvend: Castlepoint Press.

Kleyer, M., R.M. Bekker, I.C. Knevel, J.P. Bakker, K. Thompson,M. Sonnenschein, P. Poschlod, J.M. van Groenendael, et al. 2008. The LEDA Traitbase: a database of life-history traits of the Northwest European flora. *Journal of Ecology* 96: 1266-1274.

Klotz, S., I. Kühn, and W. Durka. 2002. Eine Datenbank zu biologisch-ökologischen Merkmalen der Gefäßpflanzen in Deutschland. Bonn.. Bundesamt für Naturschutz.

Kutschera, L. 1960. *Wurzelatlas mitteleuropäischer Ackerunkräuter und Kulturpflanzen*. Frankfurt am Main: DLG.

Metselaar, K., M. Sobotik, D. Haas, Q. de Jong van Lier, and L. Mommer. 2009. Rooting profiles with depth – the case of the Wurzelatlas mitteleuropäischer Ackerunkräuter und Kulturpflanzen. Vienna: International Symposium “Root Research and Applications”.

Species list and trait data for hydrophytes.

Table S4 Species list and traits of hydrophytes (n=17), modelled as separate functional group assumed to occupy primarily flowing water bodies in riparian wetlands. Two categorical traits were used: Space occupancy is derived from an index calculated as (height+lateral spread)/2 (cm), where small corresponds to <10cm, medium 100-100 cm, large >100cm); it indicates size/ biomass, or space occupancy. Body flexibility describes the degree of deformation under water flow pressure, where rigid corresponds to yielding of <45°, elastic 45-300°, and soft >300° (data from Willby et al. 2000).

Species	Occupancy Index	Body flexibility
Alisma plantago-aquatica	medium	elastic
Glyceria fluitans	medium	elastic
Hippuris vulgaris	medium	elastic
Lemna minor	small	rigid
Mentha aquatica	medium	rigid
Myosotis scorpioides	medium	rigid
Nuphar lutea	large	elastic
Nymphaea alba	large	elastic
Persicaria amphibia	large	elastic
Potamogeton natans	large	soft
Ranunculus flammula	small	elastic
Schoenoplectus lacustris	large	elastic
Sium latifolium	large	rigid
Sparganium natans	medium	elastic
Utricularia intermedia	medium	elastic
Utricularia minor	medium	elastic
Utricularia vulgaris	medium	elastic

Willby, N., V.J. Abernethy, and B.O.L. Demars. 2000. Attribute-based classification of European hydrophytes and its relationship to habitat utilization. Freshwater Biology 43:43-74

Electronic Supplementary Material – Results

Species importance for trait change (vascular plants)

Relative impact of the distributional change of each species was assessed by excluding that species from community weighted mean trait (CWMT) value calculations, and then calculating a standardized mean squared error, averaged across the NDB.

Specifically, the difference in CWMT values caused by excluding the species across NDB was calculated, squared, and divided by the number of pixels, according to

$MSE = 1/n * \sum(CWM_{all} - CWM_{reduced})^2$

MSE was then standardized to yield a contribution coefficient CC according to

$CC = \sqrt{MSE} / averageCWM_{all}$

This was done for each trait to assess the contribution of each species to CWM change in each trait. Here we report the average contribution of vascular plant species across all traits (Table S5).

Table S5 Vascular plant species contributing most to trait change across all traits across the NDB, separated for wetland type and vegetation layer, and sorted for relative importance (given by the average CC across all traits). The two most important species for change across all traits for field and shrub layers of each wetland type are highlighted in bold typeface; species that showed particularly strong contribution to change in single traits (cf. results section) are additionally highlighted in italics.

Wetland	Layer	Species	meanCC
bog	***field***	***Eriophorum vaginatum***	***0.97***
bog	***field***	***Drosera rotundifolia***	***0.83***
bog	field	Scheuchzeria palustris	0.19
bog	*field*	*Andromeda polifolia*	*0.16*
bog	field	Carex magellanica	0.15
bog	*field*	*Rhynchospora alba*	*0.09*
bog	field	Carex limosa	0.09
bog	field	Rubus chamaemorus	0.07
bog	field	Trichophorum cespitosum	0.06
bog	***shrub***	***Rhododendron tomentosum***	***2.89***
bog	***shrub***	***Vaccinium myrtillus***	***0.80***
bog	shrub	Calluna vulgaris	0.53
bog	*shrub*	*Betula nana*	*0.42*
bog	shrub	Empetrum nigrum	0.14
bog	shrub	Vaccinium uliginosum	0.11
bog	shrub	Empetrum hermaphroditum	0.08
bog	shrub	Vaccinium oxycoccos	0.07
bog	shrub	Erica tetralix	0.02
fen	***field***	***Phragmites australis***	***0.85***
fen	***field***	***Phalaris arundinacea***	***0.72***
fe*n*	*field*	*Carex lasiocarpa*	*0.37*
fen	field	Carex limosa	0.29
fen	field	Carex canescens	0.27

fen	field	Schoenus ferrugineus	0.26
fen	field	Equisetum sylvaticum	0.25
fen	field	Molinia caerulea	0.23
fen	field	Trichophorum cespitosum	0.13
fen	field	Equisetum fluviatile	0.12
fen	field	Carex magellanica	0.12
fen	field	Carex vesicaria	0.12
fen	field	Menyanthes trifoliata	0.09
fen	*field*	*Agrostis canina*	*0.08*
fen	field	Potentilla palustris	0.08
fen	field	Carex chordorrhiza	0.06
fen	field	Eriophorum angustifolium	0.06
fen	field	Viola palustris	0.05
fen	field	Carex rostrata	0.04
fen	field	Carex nigra	0.03
fen	field	Drosera intermedia	0.02
fen	field	Narthecium ossifragum	0.01
fen	***shrub***	***Salix repens***	***0.65***
fen	***shrub***	***Myrica gale***	***0.43***
fen	shrub	Calluna vulgaris	0.21
fen	shrub	Betula nana	0.20
fen	shrub	Salix lapponum	0.09
fen	shrub	Vaccinium oxycoccos	0.08
rip	***field***	***Phragmites australis***	***1.08***
rip	***field***	***Equisetum palustre***	***0.22***
rip	field	Carex rostrata	0.20
rip	field	Glyceria maxima	0.17
rip	*field*	*Agrostis stolonifera*	*0.15*
rip	*field*	*Typha latifolia*	*0.14*
rip	field	Equisetum sylvaticum	0.12
rip	field	Phalaris arundinacea	0.12
rip	*field*	*Lycopus europaeus*	*0.11*
rip	field	Scirpus sylvaticus	0.10
rip	field	Juncus filiformis	0.10
rip	field	Lysimachia thyrsiflora	0.08
rip	field	Caltha palustris	0.08
rip	field	Iris pseudacorus	0.07
rip	field	Carex vesicaria	0.07
rip	*field*	*Lysimachia vulgaris*	*0.07*
rip	field	Calamagrostis canescens	0.07
rip	field	Carex acuta	0.06
rip	field	Potentilla palustris	0.06
rip	field	Carex canescens	0.06
rip	field	Lythrum salicaria	0.06
rip	field	Cirsium palustre	0.05
rip	field	Deschampsia cespitosa	0.05
rip	field	Equisetum fluviatile	0.05
rip	field	Calla palustris	0.04
rip	field	Filipendula ulmaria	0.04
rip	field	Juncus effusus	0.03
rip	field	Carex aquatilis	0.01
rip	field	Calamagrostis purpurea	0.01
rip	***shrub***	***Salix cinerea***	***7.48***
rip	***shrub***	***Salix caprea***	***7.37***
rip	shrub	Myrica gale	1.18
rip	shrub	Salix lapponum	0.55

Moor H., K. Hylander, and J. Norberg (2015)

Predicted change in relative biomass of species (vascular plants)

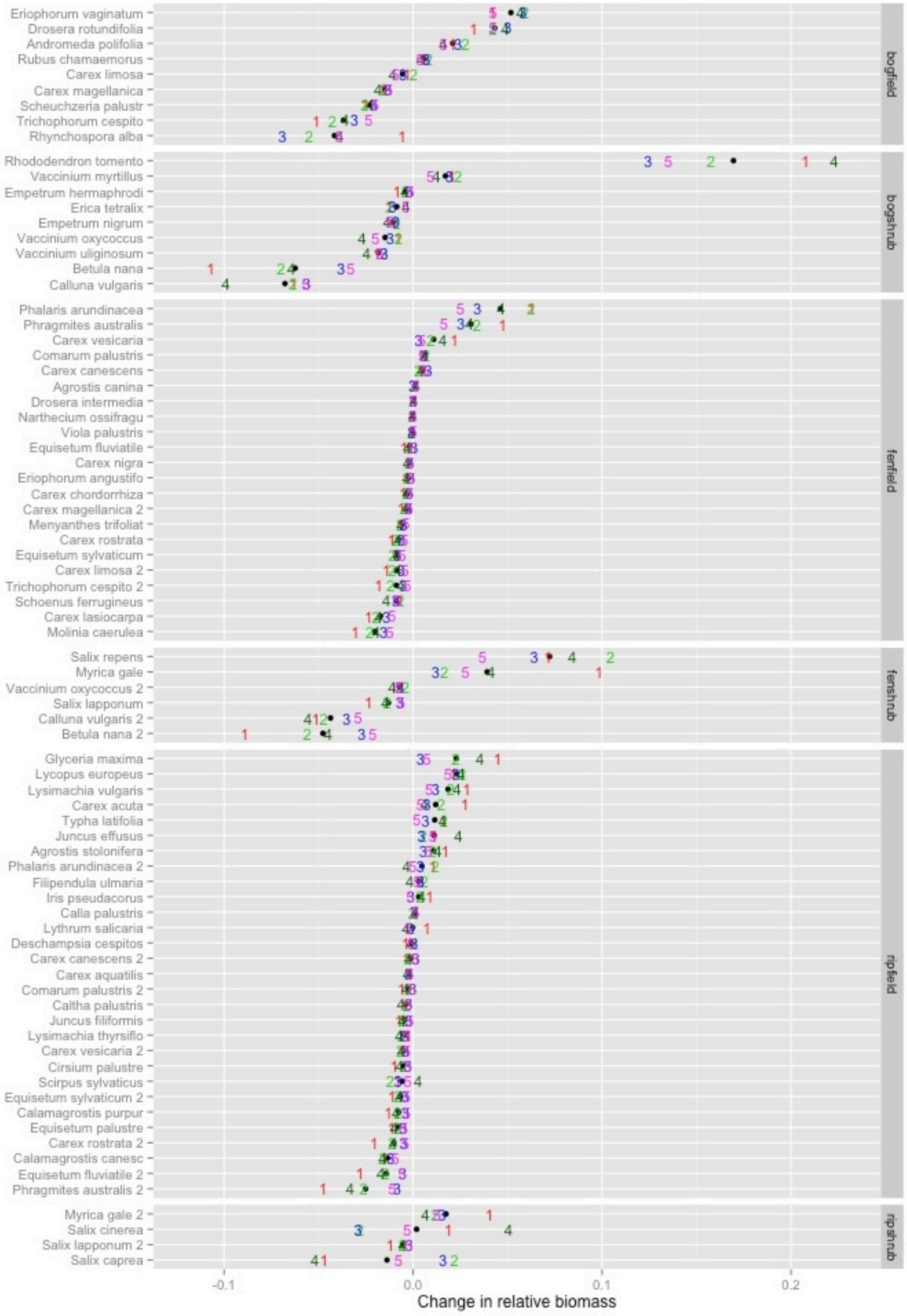

Fig. S2 (previous page) Proportional shifts in relative biomass of vascular plants in field and shrub layers of the three wetland types. Black dots indicate the overall mean across the NDB, and colored numbers show the average change in each of the five regions.

Note that changes in species proportions of community biomass are relatively larger where species lists are shorter. This drawback of our approach might be less pronounced with more extensive species lists, but we believe our species selection covers those species that collectively dominate community biomass. To resolve this issue, field data on realized relative biomass across both field and shrub layers (and potentially even mosses and trees) would be required.

Strongest change in predicted distribution of bryophytes in the NDB

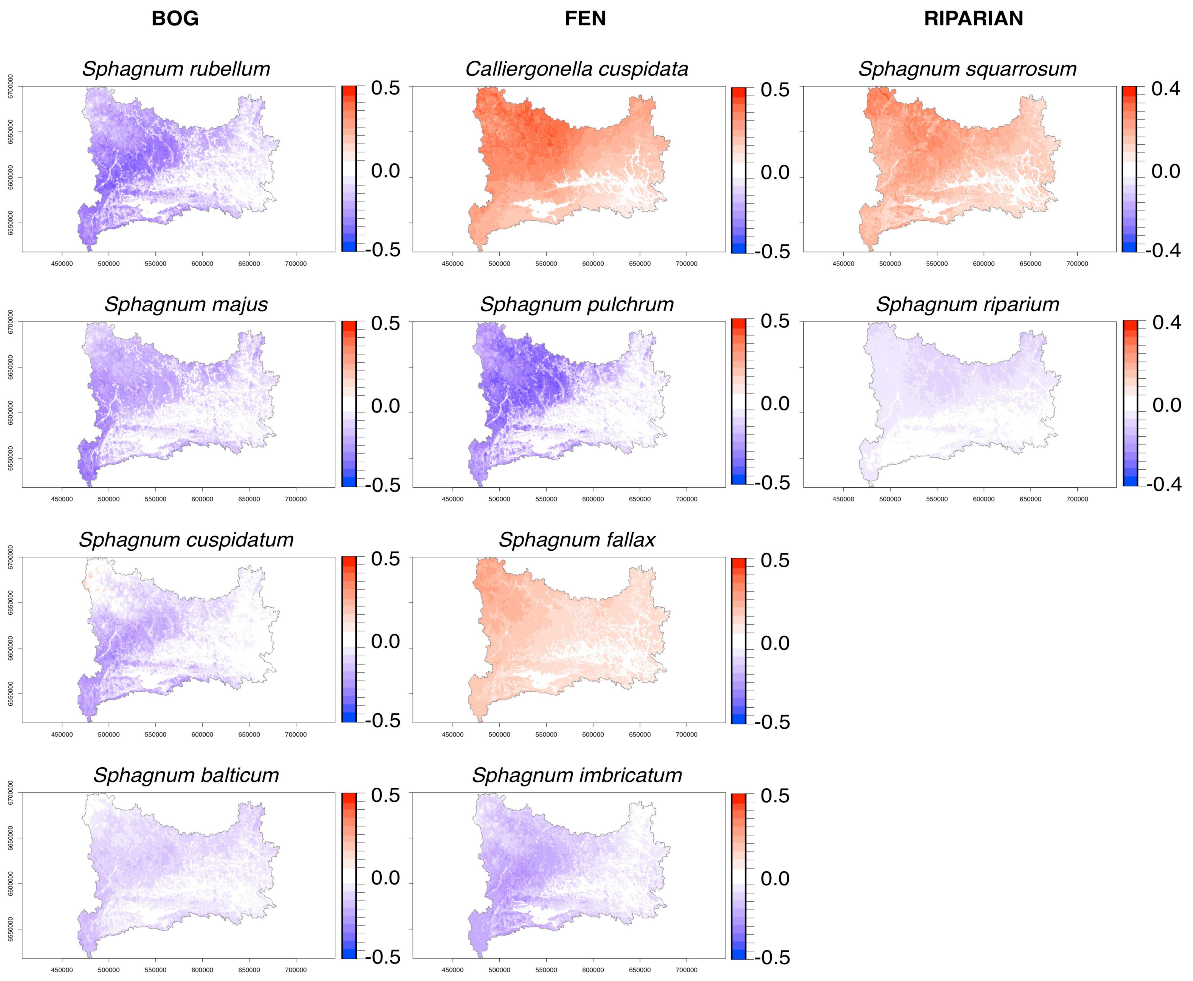

Fig. S3 Strongest predicted change in abundance distribution of bryophytes in the NDB for the three wetland types**.** Blue indicates decrease, red increase. The scale shows change in suitability relative to current suitability (between 0 and 1).

Absolute values of community weighted mean trait values, current and future predicted

Table S6 Absolute CMWT values for continuous traits specific leaf area (SLA), canopy height (CH), and root depth (RD) at the times current and future for field and shrub layers in the three wetland types, averaged in the five regions. These average values are the basis for calculation of percent change shown in figures in the main text.

Time	Trait	Wetland type	Vegetation layer	Unit	Region 1	Region 2	Region 3	Region 4	Region 5
Current	SLA	Bog	Field	$mm^2\ mg^{-1}$	16.58	16.88	17.33	17.37	17.41
Current	SLA	Bog	Shrub	$mm^2\ mg^{-1}$	12.69	12.71	12.74	12.93	12.93
Current	SLA	Fen	Field	$mm^2\ mg^{-1}$	16.68	16.89	16.98	16.83	16.96
Current	SLA	Fen	Shrub	$mm^2\ mg^{-1}$	13.37	14.09	15.06	14.99	15.51
Current	SLA	Riparian	Field	$mm^2\ mg^{-1}$	15.84	16.29	16.59	16.38	16.71
Current	SLA	Riparian	Shrub	$mm^2\ mg^{-1}$	12.56	12.66	12.75	13.28	13.01
Current	CH	Bog	Field	m	0.28	0.28	0.28	0.28	0.28
Current	CH	Bog	Shrub	m	0.64	0.68	0.70	0.66	0.69
Current	CH	Fen	Field	m	1.12	1.17	1.23	1.20	1.26
Current	CH	Fen	Shrub	m	0.92	0.96	1.00	0.97	0.99
Current	CH	Riparian	Field	m	1.11	1.08	1.07	1.09	1.08
Current	CH	Riparian	Shrub	m	3.96	4.10	4.24	4.62	4.46
Current	RD	Bog	Field	cm	69.78	68.86	67.36	67.64	67.06
Current	RD	Bog	Shrub	cm	69.63	67.69	66.30	66.98	65.94
Current	RD	Fen	Field	cm	94.00	95.98	99.31	98.21	100.65
Current	RD	Fen	Shrub	cm	106.08	112.89	122.75	120.62	126.05
Current	RD	Riparian	Field	cm	105.12	104.37	104.87	105.19	105.39
Current	RD	Riparian	Shrub	cm	184.56	187.70	191.21	203.44	197.66
Future	SLA	Bog	Field	$mm^2\ mg^{-1}$	17.22	17.66	18.24	18.24	18.19
Future	SLA	Bog	Shrub	$mm^2\ mg^{-1}$	12.78	12.88	12.87	12.87	12.91
Future	SLA	Fen	Field	$mm^2\ mg^{-1}$	16.75	16.93	17.07	17.00	17.10
Future	SLA	Fen	Shrub	$mm^2\ mg^{-1}$	14.00	15.01	15.62	15.74	15.83
Future	SLA	Riparian	Field	$mm^2\ mg^{-1}$	17.42	17.68	17.73	17.72	17.70
Future	SLA	Riparian	Shrub	$mm^2\ mg^{-1}$	12.39	12.77	12.84	13.05	12.99
Future	CH	Bog	Field	m	0.28	0.28	0.28	0.29	0.28
Future	CH	Bog	Shrub	m	0.72	0.73	0.75	0.75	0.74
Future	CH	Fen	Field	m	1.28	1.30	1.32	1.31	1.31
Future	CH	Fen	Shrub	m	1.00	1.00	1.02	1.02	1.02
Future	CH	Riparian	Field	m	1.08	1.07	1.07	1.07	1.07
Future	CH	Riparian	Shrub	m	3.77	4.16	4.28	4.45	4.42
Future	RD	Bog	Field	cm	67.74	66.38	64.58	64.76	64.68
Future	RD	Bog	Shrub	cm	64.40	64.77	65.44	65.91	65.77
Future	RD	Fen	Field	cm	99.26	100.48	101.69	101.86	102.31
Future	RD	Fen	Shrub	cm	111.32	120.28	128.31	128.30	130.01
Future	RD	Riparian	Field	cm	100.89	101.46	102.23	102.36	102.88
Future	RD	Riparian	Shrub	cm	178.50	189.57	192.48	197.74	196.55

Table S7 Absolute values for proportions of levels of the categorical traits plant functional group (PFG), Leaf persistence, clonal growth form, and mycorrhizal association modeled for present, for field and shrub layers in the three wetland types, averaged in the five regions. These average values are the basis for calculation of percent change shown in figures in the main text.

Time	Trait	Wetland type	Vegetation layer	Trait level	Region 1	Region 2	Region 3	Region 4	Region 5
Current	PFG	Bog	Field	herbaceous	0.22	0.24	0.28	0.29	0.32
Current	PFG	Fen	Field	graminoid	0.80	0.80	0.81	0.81	0.82
Current	PFG	Fen	Field	herbaceous	0.07	0.07	0.07	0.07	0.07
Current	PFG	Fen	Field	pteridophyte	0.13	0.12	0.11	0.12	0.12
Current	PFG	Riparian	Field	graminoid	0.66	0.66	0.66	0.64	0.65
Current	PFG	Riparian	Field	herbaceous	0.24	0.26	0.28	0.28	0.29
Current	PFG	Riparian	Field	pteridophyte	0.10	0.08	0.06	0.08	0.06
Current	Leaf persistence	Bog	Field	persistent	0.50	0.53	0.57	0.59	0.60
Current	Leaf persistence	Bog	Field	summergreen	0.50	0.47	0.43	0.41	0.40
Current	Leaf persistence	Bog	Shrub	persistent	0.70	0.74	0.78	0.73	0.77
Current	Leaf persistence	Bog	Shrub	summergreen	0.30	0.26	0.22	0.27	0.23
Current	Leaf persistence	Fen	Field	persistent	0.22	0.18	0.15	0.17	0.14
Current	Leaf persistence	Fen	Field	summergreen	0.78	0.82	0.85	0.83	0.86
Current	Leaf persistence	Fen	Shrub	persistent	0.35	0.31	0.24	0.28	0.23
Current	Leaf persistence	Fen	Shrub	summergreen	0.65	0.69	0.76	0.72	0.77

Current	Leaf persistence	Riparian	Field	persistent	0.24	0.23	0.22	0.19	0.20
Current	Leaf persistence	Riparian	Field	summergreen	0.76	0.77	0.78	0.81	0.80
Current	Leaf persistence	Riparian	Shrub	summergreen	1.00	1.00	1.00	1.00	1.00
Current	Clonal growth form	Bog	Field	Rhizomatous	0.61	0.61	0.58	0.57	0.58
Current	Clonal growth form	Bog	Field	Non-clonal	0.04	0.06	0.08	0.08	0.09
Current	Clonal growth form	Bog	Field	Rhizomatous	0.34	0.34	0.34	0.35	0.33
Current	Clonal growth form	Fen	Field	Rhizomatous	0.87	0.90	0.92	0.91	0.92
Current	Clonal growth form	Fen	Field	Non-clonal	0.00	0.00	0.00	0.00	0.00
Current	Clonal growth form	Fen	Field	Rhizomatous	0.13	0.10	0.08	0.09	0.07
Current	Clonal growth form	Riparian	Field	Rhizomatous	0.82	0.82	0.82	0.83	0.83
Current	Clonal growth form	Riparian	Field	Non-clonal	0.09	0.09	0.09	0.10	0.10
Current	Clonal growth form	Riparian	Field	Rhizomatous	0.09	0.08	0.08	0.06	0.07
Current	Mycorrhiza	Bog	Field	Obligatory	0.13	0.14	0.15	0.16	0.18
Current	Mycorrhiza	Bog	Field	non-myc.	0.39	0.37	0.33	0.31	0.30
Current	Mycorrhiza	Bog	Field	Facultative	0.48	0.49	0.52	0.53	0.52
Current	Mycorrhiza	Bog	Shrub	Obligatory	1.00	1.00	1.00	1.00	1.00
Current	Mycorrhiza	Bog	Field	graminoid	0.78	0.76	0.72	0.71	0.68
Current	Mycorrhiza	Fen	Field	Obligatory	0.09	0.07	0.06	0.07	0.06
Current	Mycorrhiza	Fen	Field	non-myc.	0.22	0.21	0.18	0.19	0.17
Current	Mycorrhiza	Fen	Field	Facultative	0.69	0.72	0.76	0.74	0.77
Current	Mycorrhiza	Fen	Shrub	Obligatory	0.69	0.67	0.68	0.74	0.72
Current	Mycorrhiza	Fen	Shrub	Facultative	0.31	0.33	0.32	0.26	0.28
Current	Mycorrhiza	Riparian	Field	non-myc.	0.25	0.23	0.19	0.22	0.18
Current	Mycorrhiza	Riparian	Field	Facultative	0.75	0.77	0.81	0.78	0.82
Current	Mycorrhiza	Riparian	Shrub	Obligatory	0.84	0.86	0.89	0.90	0.91
Current	Mycorrhiza	Riparian	Shrub	Facultative	0.16	0.14	0.11	0.10	0.09
Future	PFG	Bog	Field	herbaceous	0.28	0.32	0.36	0.36	0.38
Future	PFG	Fen	Field	graminoid	0.82	0.82	0.82	0.82	0.82
Future	PFG	Fen	Field	herbaceous	0.07	0.07	0.07	0.07	0.07
Future	PFG	Fen	Field	pteridophyte	0.11	0.11	0.11	0.11	0.11
Future	PFG	Riparian	Field	graminoid	0.66	0.65	0.65	0.65	0.65
Future	PFG	Riparian	Field	herbaceous	0.29	0.30	0.31	0.30	0.31
Future	PFG	Riparian	Field	pteridophyte	0.05	0.05	0.04	0.05	0.04
Future	Leaf persistence	Bog	Field	persistent	0.56	0.61	0.64	0.65	0.65
Future	Leaf persistence	Bog	Field	summergreen	0.44	0.39	0.36	0.35	0.35
Future	Leaf persistence	Bog	Shrub	persistent	0.80	0.81	0.81	0.81	0.81
Future	Leaf persistence	Bog	Shrub	Summergreen	0.20	0.19	0.19	0.19	0.19
Future	Leaf persistence	Fen	Field	persistent	0.15	0.13	0.12	0.12	0.11
Future	Leaf persistence	Fen	Field	summergreen	0.85	0.87	0.88	0.88	0.89
Future	Leaf persistence	Fen	Shrub	persistent	0.30	0.25	0.20	0.21	0.20
Future	Leaf persistence	Fen	Shrub	Summergreen	0.70	0.75	0.80	0.79	0.80
Future	Leaf persistence	Riparian	Field	persistent	0.21	0.21	0.21	0.20	0.20
Future	Leaf persistence	Riparian	Field	summergreen	0.79	0.79	0.79	0.80	0.80
Future	Leaf persistence	Riparian	Shrub	Summergreen	1.00	1.00	1.00	1.00	1.00
Future	Clonal growth form	Bog	Field	Rhizomatous	0.59	0.55	0.50	0.50	0.52
Future	Clonal growth form	Bog	Field	Non-clonal	0.08	0.10	0.13	0.13	0.13
Future	Clonal growth form	Bog	Field	Rhizomatous	0.33	0.35	0.37	0.37	0.35
Future	Clonal growth form	Fen	Field	Rhizomatous	0.92	0.94	0.95	0.95	0.95
Future	Clonal growth form	Fen	Field	Non-clonal	0.00	0.00	0.00	0.00	0.00
Future	Clonal growth form	Fen	Field	Rhizomatous	0.08	0.06	0.05	0.05	0.05
Future	Clonal growth form	Riparian	Field	Rhizomatous	0.82	0.83	0.82	0.83	0.82
Future	Clonal growth form	Riparian	Field	Non-clonal	0.08	0.08	0.09	0.08	0.09
Future	Clonal growth form	Riparian	Field	Rhizomatous	0.09	0.08	0.08	0.08	0.08
Future	Mycorrhiza	Bog	Field	Obligatory	0.15	0.16	0.17	0.17	0.20
Future	Mycorrhiza	Bog	Field	non-myc.	0.36	0.29	0.24	0.24	0.24
Future	Mycorrhiza	Bog	Field	Facultative	0.49	0.54	0.59	0.59	0.57
Future	Mycorrhiza	Bog	Shrub	Obligatory	1.00	1.00	1.00	1.00	1.00
Future	Mycorrhiza	Bog	Field	graminoid	0.72	0.68	0.64	0.64	0.62
Future	Mycorrhiza	Fen	Field	Obligatory	0.06	0.05	0.05	0.05	0.05
Future	Mycorrhiza	Fen	Field	non-myc.	0.19	0.19	0.17	0.18	0.17
Future	Mycorrhiza	Fen	Field	Facultative	0.75	0.77	0.78	0.78	0.79
Future	Mycorrhiza	Fen	Shrub	Obligatory	0.59	0.65	0.67	0.69	0.69
Future	Mycorrhiza	Fen	Shrub	Facultative	0.41	0.35	0.33	0.31	0.31
Future	Mycorrhiza	Riparian	Field	non-myc.	0.20	0.19	0.17	0.18	0.17
Future	Mycorrhiza	Riparian	Field	Facultative	0.80	0.81	0.83	0.82	0.83
Future	Mycorrhiza	Riparian	Shrub	Obligatory	0.80	0.85	0.88	0.89	0.89
Future	Mycorrhiza	Riparian	Shrub	Facultative	0.20	0.15	0.12	0.11	0.11
